# OsbZIP47 Is an Integrator for Meristem Regulators During Rice Plant Growth and Development

**DOI:** 10.3389/fpls.2022.865928

**Published:** 2022-04-13

**Authors:** Sandhan Prakash, Rashmi Rai, Mohamed Zamzam, Owais Ahmad, Raghavaram Peesapati, Usha Vijayraghavan

**Affiliations:** Department of Microbiology and Cell Biology, Indian Institute of Science, Bengaluru, India

**Keywords:** *Oryza sativa*, shoot apical meristem, panicle, floret, *PERIANTHIA*, *FASCIATED EAR4*, OsMADS1

## Abstract

Stem cell homeostasis by the WUSCHEL–CLAVATA (WUS-CLV) feedback loop is generally conserved across species; however, its links with other meristem regulators can be species-specific, rice being an example. We characterized the role of rice *OsbZIP47* in vegetative and reproductive development. The knockdown (KD) transgenics showed meristem size abnormality and defects in developmental progression. The size of the shoot apical meristem (SAM) in 25-day *OsbZIP47KD* plants was increased as compared to the wild-type (WT). Inflorescence of KD plants showed reduced rachis length, number of primary branches, and spikelets. Florets had defects in the second and third whorl organs and increased organ number. *OsbZIP47KD* SAM and panicles had abnormal expression for CLAVATA peptide-like signaling genes, such as *FON2-LIKE CLE PROTEIN1* (*FCP1*), *FLORAL ORGAN NUMBER 2* (*FON2*), and hormone pathway genes, such as cytokinin (CK) *ISOPENTEYLTRANSFERASE1* (*OsIPT1*), *ISOPENTEYLTRANSFERASE 8* (*OsIPT8*), auxin biosynthesis *OsYUCCA6, OsYUCCA*7 and gibberellic acid (*G*A) biosynthesis genes, such as *GRAIN NUMBER PER PANICLE1* (*GNP1/OsGA20OX1*) and *SHORTENED BASAL INTERNODE* (*SBI/OsGA2ox4*). The effects on *ABBERANT PANICLE ORGANIZATION1* (*APO1*), *OsMADS16*, and *DROOPING LEAF* (*DL*) relate to the second and third whorl floret phenotypes in *OsbZIP47KD*. Protein interaction assays showed OsbZIP47 partnerships with RICE HOMEOBOX1 (OSH1), RICE FLORICULA/LEAFY (RFL), and OsMADS1 transcription factors. The meta-analysis of KD panicle transcriptomes in *OsbZIP47KD*, *OsMADS1KD*, and *RFLKD* transgenics, combined with global OSH1 binding sites divulge potential targets coregulated by OsbZIP47, OsMADS1, OSH1, and RFL. Further, we demonstrate that OsbZIP47 redox status affects its DNA binding affinity to a *cis* element in *FCP1*, a target locus. Taken together, we provide insights on *OsbZIP47* roles in SAM development, inflorescence branching, and floret development.

## Introduction

The post-embryonic shoot development in flowering plants depends on the balance between stem cell renewal in the central zone of above ground meristems and the adoption of specific differentiation programs in cells from the peripheral zone. The genetic framework of the basic WUSCHEL–CLAVATA (WUS-CLV) pathway for meristem maintenance is largely conserved in monocots and dicots, yet some functional differences are reported among cereal grass models, such as maize and rice. In maize, functions for *TASSEL DWARF1* (*TD1, CLV1* ortholog) and *FASCIATED EAR2* (*FEA2, CLV2* ortholog) in the shoot apical meristem (SAM) are not obvious in the respective mutants, yet mutants in these genes have significant and somewhat differential effects on the female *vs.* male inflorescence meristems (IMs) (Bommert et al., 2005; Dodsworth, 2009; Pautler et al., 2013; Chongloi et al., 2019). In rice, *FLORAL ORGAN NUMBER 1* (*FON1*) is the *CLV1* ortholog, while *FON2/FON4*, *FON2 SPARE1* (*FOS1*) and *FON2-LIKE CLE PROTEIN1* (*FCP1*) encode CLV3 peptide paralogs. FON2 signaling through FON1 majorly regulates the homeostasis of IMs whereas FCP1 triggered signaling regulates SAM through effects on *WUSCHEL RELATED HOMEOBOX* (*OsWOX4*), functionally related to *AtWUS* (Nagasawa et al., 1996; Suzaki et al., 2004, 2006; Ohmori et al., 2013). These tissue-specific effects of maize *TD1*, *FEA2*, and of rice *CLV3-like* genes exemplify species-specific innovations in signaling components of this core meristem regulatory circuit. In the SAM of Arabidopsis, AtWUS activates *SHOOT MERISTEMLESS (STM*), and both directly regulate *CLV3* expression to maintain constant stem cell number (Su et al., 2020). Integration of CLV-WUS pathway with the roles of class I *KNOTTED-1-LIKE HOMEOBOX* (*KNOX*) genes, Arabidopsis *STM*, rice *HOMEOBOX1* (*OSH1*), and maize *KNOTTED1* (*KN1*) in meristem maintenance is conserved across species (Vollbrecht et al., 2000; Brand et al., 2002; Tsuda et al., 2011). Similarly, the interlinking of WUS-CLV pathway with phytohormone-based meristem control, by cytokinin (CK), auxin (IAA/AUX), gibberellin (GA), brassinosteriod (BR) actions, is also conserved (Kurakawa et al., 2007; Gordon et al., 2009; Lee et al., 2009; Zhao et al., 2010; Yamaki et al., 2011; Somssich et al., 2016). OSH1 positively autoregulates itself directly by binding to evolutionarily conserved *cis*-elements within its locus (Tsuda et al., 2011). Further, OSH1 induces the expression of CK biosynthesis genes, such as *ADENOSINE PHOSPHATE ISOPENTENYL TRANSFERASE 2* (*OsIPT2*) and *OsIPT3* (Sakamoto et al., 2006). Interestingly, CK treatment of callus activates the transcription of rice *KNOX* genes (Tsuda et al., 2011). AtWUS and OsWOX4 also regulate CK signaling in Arabidopsis and rice, respectively (Leibfried et al., 2005; Ohmori et al., 2013). In the floral meristem center, the timing of the termination of stem cell activity is co-incident with carpel/ovule specification. This creates a determinate floral meristem for normal reproduction. Meristem termination is mediated by the concerted activity of floral organ identity genes (Class A, C, and E) whose regulatory effects on *WUS*-*CLV* and *WUS*-*KNOX* pathway genes operate in both monocots and eudicots [reviewed by Tanaka et al. (2013), Callens et al. (2018), and Chongloi et al. (2019)]. These genes are in turn spatially and temporally regulated. For example, Arabidopsis *LEAFY* (*LFY*) directly activates *APETALA1* (*AP1*) and *WUS* (Lenhard et al., 2001; Lohmann et al., 2001) while repressing the shoot meristem identity gene, *TERMINAL FLOWER1* (*TFL1*) (Moyroud et al., 2009, 2010). Furthermore, in young floral meristems, *LFY* together with *UNUSUAL FLORAL ORGAN* (*UFO*) and *WUS* activate *APETALA3* (*AP3*) and *AGAMOUS* (*AG*) gene expression in the third and fourth whorls of the developing meristem (Parcy et al., 1998; Busch et al., 1999; Wagner et al., 1999; Lenhard et al., 2001; Lohmann et al., 2001). In the later stages of floral meristem development, AG directly activates *KNUCKLES* (*KNU*) which leads to the repression of *WUS* by the recruitment of Polycomb group (PcG) chromatin modifiers (Ming and Ma, 2009; Sun et al., 2009, 2014; Liu et al., 2011; Zhang, 2014). Aside from LFY, the *AG* expression is also influenced by *PERIANTHIA* (*PAN*) that encodes a bZIP class TF, whose orthologs are *Oryza sativa basic LEUCINE ZIPPER* 47 (*OsbZIP47*) and maize *FASCIATED EAR4* (*ZmFEA4*). Floral meristem size and organ patterning defects in the Arabidopsis *pan-3 lfy-31* double the mutant, and in transgenics with modified PAN fusion proteins (repressive *vs*. activated forms) show roles of *AtPAN* in floral determinacy, meristem size, and floral organ patterning (Running and Meyerowitz, 1996; Chuang et al., 1999; Das et al., 2009; Maier et al., 2009, 2011). Maize *ZmFEA4* activates the expression of genes involved in AUX pathway and lateral organ differentiation and also regulates both SAM and IM size homeostasis (Pautler et al., 2015). Unlike Arabidopsis and maize, *OsbZIP47* (LOC_Os06g15480) is not well-characterized, and its interacting partners are largely unknown. Further, how inflorescence BM identity and transition regulators intersect with the two meristem maintenance pathways (CLV-WUS and KNOX1) is not much explored in rice. Here, functional characterization of *OsbZIP47* by RNA interference (dsRNAi)-based knockdown (KD) and identification of some meristem regulators, such as OsbZIP47 interacting partners sheds light on its role in meristem size and meristem developmental progression. Further, our transcriptome and meta-analysis uncovered downstream pathways that can be co-regulated by OsbZIP47 and OSH1, OsMADS1, or RFL.

## Materials and Methods

### Plasmid Constructs Generation and Rice Transformation

For siRNA (interference) mediated KD of endogenous *OsbZIP47*, a gene-specific 226bp 3′UTR DNA fragment was cloned in the sense and in the antisense orientation in pBluescript vector, and were separated by a 270-bp linker. Subsequently, the insert in recombinant pBluescript was re-cloned in the binary rice expression vector, pUN downstream to the maize ubiquitin promoter for the expression of *OsbZIP47* hairpin RNAs (Supplementary Figure 1; Prasad et al., 2001). For the over-expression of *OsbZIP47*, the full length cDNA was cloned at *Bam*HI (blunted)-*Kpn*I sites in the pUN vector to create *pUbi*:*OsbZIP47* (Supplementary Figure 4). These constructs for KD and over-expression of *OsbZIP47* were transformed into the *Agrobacterium tumefaciens* strain, LBA4404 and then co-cultivated with embryogenic calli from TP309 WT (*O. Sativa* var *japonica*) seeds as described by Prasad et al. (2001). Transgenic plants, dsRNAi *OsbZIP47* and Ox-*OsbZIP47*, were grown in IISc, Bangalore, Green house condition, approximately at 27°C during the months of January–May and July–October always with wild-type (WT) controls.

### Phenotypic Characterization of Knockdown Transgenic

The transgenic plants, such as dsRNAi *OsbZIP47* and Ox-*OsbZIP47* were selected on half-strength MS medium containing 50 mg L^–1^ of hygromycin. Phenotypic analysis was done with T3 dsRNAi *OsbZIP47* transgenics and T1 Ox-*OsbZIP47* lines. Eosin-hematoxylin stained 25 DAG seedling tissue sections (7 μm, Lecia microtome; RM2045) were imaged by Apotome2 Zeiss. The cell size in the SAM was measured by ImageJ. The seedling height was measured at age eight DAG. Adult plant height, lamina joint angle, panicle length, branch characteristics, and spikelet numbers were measured after panicle booting. Pre-anthesis florets, in panicles prior to emergence from the flag leaf, were imaged using Leica Wild M3Z stereomicroscope.

### RNA-Sequencing and RT-qPCR

Next Generation Sequencing (NGS) of RNA from *OsbZIP47* KD panicles (0.1–0.5 cm) was done for two biological replicates with matched WT panicles as controls. The total RNA was extracted using Trizol Reagent (Sigma) according to manufacturer’s instructions. About 1 μg of total RNA was used for library preparation using rRNA depletion-based NEB Next UltraII RNA kit. The NGS was performed using Illumina Hi-Seq, pair-end 2 × 150 bp chemistry. After quality check (using FastQC and multiQC software), the reads were mapped against indexed *O. sativa* ssp. *japonica* cv. reference genome (RAP-DB)^1^ using STAR2 (v2.5.3a). Further, differential gene expression (DGE) of read counts between WT and transgenics were computed using edgeR (v3.28.0) package with the absolute log2 fold change ≥ 1 with *p*-value ≤ 0.05. For real-time qPCR experiments, oligo(dT)-primed cDNAs were synthesized using 2 ug of total RNA with MMLV (reverse transcriptase, NEB). The qRT-PCR reactions were set up with 50–70 ng of cDNA, 250 nM gene-specific primers, and FastStart Universal Sybr Green Master (Rox) mix (Roche) in CFX384 real-time system (Biorad) or Applied Biosystems ViiA 7 system. Fold change in the transcript levels of deregulated genes was calculated as a difference in cycle threshold value between transgenic and wild type. To obtain normalized threshold value (ΔΔCt), first ΔCt value was calculated by subtracting the Ct value for internal control; *Ubiquitin5*, from the Ct value for each gene of interest (Gene Ct-Ubi5 Ct). Then ΔΔCt was calculated by subtracting the WT ΔCt value from the ΔCt value obtained from the transgenic tissue. The fold-change was calculated as 2^^–(ΔΔCt)^. Primers used and their sequences are listed in Supplementary Table 2. The RNA sequencing raw data files used in this study have been deposited to Gene Expression Omnibus (GEO) database under the accession number GSE196747.

### RNA *in situ* Hybridization

To generate *OsbZIP47* riboprobes, a gene-specific 226bp DNA fragment from 3′UTR (1329-1555 bp) was PCR-amplified and cloned in the pBluescript KS + vector. Sense and antisense Digoxigenin-labeled (DIG-UTP, Roche) riboprobes were prepared by *in vitro* transcription using T3 and T7 RNA polymerases (NEB), respectively. Tissue processing and probe hybridizations was done according to the study by Prasad et al. (2005). Signal was developed using anti-digoxygenin-alkaline phosphatase (AP) conjugated antibodies (Roche) and 5-Bromo-4-chloro-3-indolyl phosphate (BCIP)-nitro blue tetrazolium (NBT) chromogenic substrates (Roche). Images were captured by Apotome2 Zeiss microscope system.

### Bacterial Expression of OsbZIP47 Full-Length Protein and Studies of Oligomeric Status

For OsbZIP47 protein expression and purification from bacteria, *OsbZIP47* full-length (FL) CDS was cloned in the pET32a vector. Thioredoxin-His-tagged OsbZIP47FL was expressed from Rosetta (DE3) bacterial strain induced with 0.2 mM of isopropyl β- *d*-1-thiogalactopyranoside (IPTG) for 3 h at 37°C. Oligomeric states of OsbZIP47 protein was determined by analytical size-exclusion chromatography (SEC) performed at 4*^o^*C on a Superdex 200 increase column Cytiva (Formerly, GE Healthcare Life Sciences), Marlborough, United States pre-equilibrated with a buffer (25 mM of sodium phosphate (pH 7.4), 100 mM of NaCl, and 5% of glycerol). Approximately, 400 μg of protein, (∼2 mg/ml) was injected into AKTA purifiers Cytiva (Formerly, GE Healthcare Life Sciences), Marlborough, United States connected to the column. The flow rate was maintained at 0.3 ml/min and the protein elution profile was at 220 nm. The molecular weight was calculated using a standard plot. Molecular weight was calculated using the equation: Y = −0.602X + 4.6036, where Y = V_*e*_/V_*o*_ (V_*e*_ = Elution volume; V_*o*_ = Void volume) and X = Log of molecular weight in Dalton.

### Electrophoretic Mobility Shift Assays

*Escherichia coli* rosetta (DE3) bacterial lysates with the Trx-His-OsbZIP47 FL was prepared in a buffer: 10mM of HEPES-KOH, pH 7.8, 50mM of NaCl, 0.5% of Non-idet P-40, 0.5 mM of EDTA, 1 mM of MgCl2, 10% of glycerol, 0.5 mM of DTT, and 1x protease inhibitor cocktail (Sigma). About 1–4 μl of lysate was incubated with 5’end P^32^ labeled DNA oligonucleotide probes for 30 min at 4°C in 1× EMSA buffer (20 mM of HEPES-KOH pH 7.8, 100 mM of KCl, 2 mM of DTT, 1 mM of ETDA, 0.1% of BSA, 10 ng of Herring sperm DNA, 10% of glycerol, 1× protease inhibitor cocktail) in 15 μl reactions. After binding, the reaction constituents were resolved on an 8% of native-PAGE gel in 0.5× Tris-borate EDTA (TBE) buffer at room temperature. Gel autoradiography was done in a phoshorimager (GE; Typhoon FLA 9500). The DNA probe sequences are listed in Supplementary Table 2.

### Microscale Thermophoresis Assay

*Escherichia coli* rosetta (DE3)-expressed proteins (Trx-His-OsbZIP47 FL and Trx-His; Supplementary Figure 9) were added to the buffer: 10mM of HEPES-KOH pH 8.0, 50 mM of NaCl, 0.5% of TWEEN-20, 0.5 mM of EDTA, 1 mM of MgCl_2_, 10% of glycerol, 2 mM of beta mercaptoethanol, and 1 mM of PMSF. About 10 mM of each protein was labeled with 5 mM of Red-NHS 2nd Generation primary amine labeling dye (NanoTemper GmbH, Cat# MO-L011), and then eluted in the buffer: 10 mM of HEPES-KOH pH 8.0, 50 mM of NaCl, 0.5% of TWEEN-20, 0.5 mM of EDTA, 1 mM of MgCl_2_, 10% of glycerol, 1X PIC (ROSCHE), and a reducing/oxidizing agent (20 mM of DTT/1 mM of oxidized glutathione). The labeled protein was incubated with dsOsFCP1 oligonucleotides which was serially diluted from 100 μM to 3.05 nM in 16 steps and fluorescence was measured using Monolith NT.115Pico (NanoTemper GmbH). The excitation power was varied between 1 and 50% to obtain measurable fluorescence signal. The MST power was varied between medium to high, to achieve high signal to noise ratio. For OsbZIP47 FL, the initial fluorescence was measured which is indicative of rapid binding. For Trx-His tag, the response evaluation was done at default on time. MO Control v1.6.1 (NanoTemper GmbH) and MO Affinity Analysis v2.6 (NanoTemper GmbH) were used for the analysis.

### Yeast Two-Hybrid Assays

The full length CDS of *OsbZIP47* was amplified from KOME clone, AK109719 using gene-specific primers, cloned into pBSKS vector and validated by restriction digestions and Sanger sequencing. The CDS was subsequently cloned into yeast two hybrid vectors, pGBDUC1 and pGADC1. Similarly, all the CDS fragments that would encode prey proteins, such as OsMADS1, OsETTIN1/2, RFL, OSH1, OsMADS15, OsMADS2, OSH15, and OsMADS15 were PCR-amplified from either KOME cDNA clones or from cDNA made from panicle tissue RNA, and subcloned into pGBDUC1 and pGADC-1 vectors. The bait clone, pGBDUC1 OsbZIP47 and indicated prey recombinants in pGADC1 were co-transformed into the yeast, pJ69-4A yeast two-hybrid (Y2H) strain (James et al., 1996). Transformants were selected on synthetic drop out media lacking leucine and uracil. Protein interactions were assessed in at least five purified transformants by serial dilution spotting of broth cultures onto SD/-Leu-Ura-His plates supplemented with 10 mM of 3AT and by the ONPG assay (Supplementary Materials and Methods).

### Bimolecular Fluorescence Complementation Assays

The *OsbZIP47* cDNA with a truncated C domain (amino acid 199–385) was cloned into pSPYCE (M) (*C*-terminal fusion) and pSPYNE (R) 173 (*N*-terminal fusion) bimolecular fluorescence complementation (BiFC) vectors (Waadt et al., 2008). Similarly, the full-length CDS encoding prey proteins, such as OsMADS1, OsETTIN 2, RFL, OSH1, and OsMADS15 were subcloned into pSPYNE (R) 173 vector. Six combinations of cEYFP and nEYFP fusions, including positive and negative controls, were transiently co-expressed in onion (*Allium cepa*) epidermal cells by *Agrobacterium. tumefaciens* (C58C1) infiltration as described by Xu et al. (2014). Co-transformed tissues were incubated at 25°C in dark for 48 h before being assayed for YFP activity. Fluorescence images were screened using a confocal laser microscope (Zeiss LSM880, Airyscan) with 2AU 480 nm excitation and 520 nm emission for the detection of YFP signal.

### Meta-Analysis

The published transcriptome datasets in dsRNAi*OsMADS1* and dsRNAi*RFL* panicles were adopted in this study to compare them with that of *OsbZIP47KD* transcriptome dataset. The differentially expressed genes (DEGs) from each dataset was taken up for pair-wise comparison to identify unique, or commonly (upregulated, or downregulated) downstream genes. The deregulated genes were also corelated with the published data on OSH1 genome-wide binding (Supplementary Materials and Methods). To align genes from the diverse datasets, i.e., transcriptomes downstream to *OsMADS1*, *RFL*, and genes bound by OSH1 for meta-analysis and for GO enrichment analyses, the gene IDs as per RAP-dB (see text footnote 1) were converted to their corresponding gene ID in MSU-TIGR v7.^2^ After this curation, among the 2,800 RAP-dB ID genes, only 2,210 genes were also annotated in MSU-TIGR v7. The list of RAP-dB gene IDs and their corresponding MSU v7 LOC_IDs are presented in Supplementary Dataset 1.

## Results

### *OsbZIP47* Knockdown Plants Have Enlarged Shoot Apical Meristem Size

**FIGURE 1:**
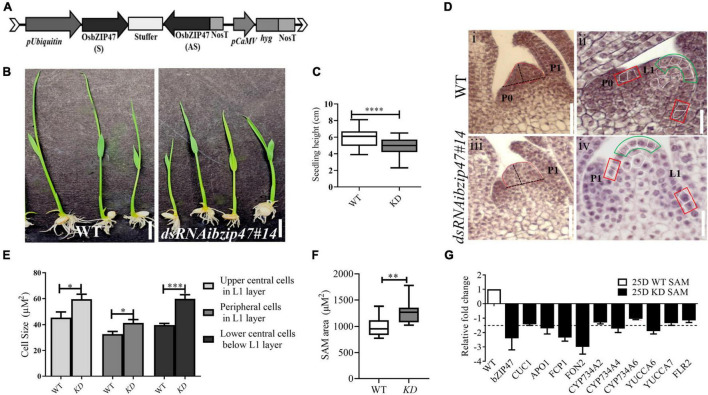
*OsbZIP47* knockdown (KD) vegetative phenotypes. **(A)** Schematic representation of *dsRNAiOsbZIP47* transgene T-DNA segment. **(B)** Seedling height of 8-day *OsbZIP47KD* (*dsRNAibZIP47#14*) plants is shorter than that of wild-type (WT) plants. Scale bar = 1 cm. **(C)** Seedling height data shown as mean ± s.d., Student’s *t-*tests, ****P < 0.0001, *n* = 30. **(D)** Representative histological sections of SAMs from WT and *dsRNAibZIP47#14*. SAM area in 25 days after germination (DAG) seedlings marked by red outline in panels **(i,iii)**. SAM height and width are marked by dashed black lines. Measured cells are marked with white outlines in panels ii and iv. Scale bar in panes **(i,iii)** are of 50 μM, and panels **(ii,iv)** are of 20 μM. **(E)** Comparison of L1 cell size in the central, and peripheral cells, and in the lower central cells underlying the central L1 layer. The statistical analysis of cell size, for cells in different regions of the meristem, was derived from 10 to 12 different sections. Data is shown as mean ± s.d. Student’s *t-*tests. *P < 0.05, ***P < 0.001. **(F)** Comparison of SAM area in *OsbZIP47KD* (*dsRNAibZIP47#14*) *vs.* WT in 25 DAG seedlings. Data is shown as mean ± s.d. Student’s *t* tests, **P < 0.01, *n* = 10. **(G)** RT-qPCR analyses of *CUC1, APO1, FCP1, FON2, CYP734A2, CYP734A4, CYP734A6, YUCCA6, DRUS2/FLR2*, and *FON2* transcripts in SAM from 25 DAG seedlings. Fold-change values were determined by comparing the normalized expression levels in *OsbZIP47KD* plants to WT plants.

To investigate the developmental roles of *OsbZIP47*, we generated thirteen independent *OsbZIP47* KD transgenics (*OsbZIP47KD*) by RNA interference (dsRNAi, Figure 1A) specific to a unique region of *OsbZIP47* 3′UTR. Based on the degree of KD and seed viability in primary T0 transgenic lines, we chose two lines; *OsbZIP47K*D line #10 and *OsbZIP47K*D line #14 for detailed phenotypic analysis in T3 generation. As a representative, phenotypic data from the *OsbZIP47K*D line #14 is further discussed here. In pooled panicle tissues (0.1–0.5 cm) from this line, qRT-PCR showed approximately 24-fold downregulation of the endogenous *OsbZIP47* transcripts (Supplementary Figure 1). The earliest phenotype noted was the seedling height at 8 days after germination (DAG), which was significantly reduced in *OsbZIP47KD* as compared to the WT (Figures 1B,C and Supplementary Table 1). This observation led us to examine SAM in the histological sections of seedlings aged 25 days after germination (DAG) from both WT and *OsbZIP47KD* plants. First, we examined SAM size and found that SAM area was increased as compared to WT (Figures 1D,F). Consistent with SAM enlargement in *OsbZIP47KD* plants, SAM width and height showed significant and marginal increase, respectively (Supplementary Table 1). To understand the cellular differences that underlie meristem size abnormalities, the cell size of L1 layer in the upper central and the peripheral zone of the meristem and the internal cells underlying the L1 layer were measured. Intriguingly, the size of these cells was increased as compared to WT (Figure 1E). Further, the spatial distribution of dividing cells in 25DAG SAMs was assessed by RNA *in situ* hybridization for the cell cycle S-phase marker, HISTONE4 (H4) (Supplementary Figure 2). Compared to WT, the overall H4 transcript signal in the median longitudinal SAM sections of *OsbZIP47KD* seedlings was higher. Altogether, these results suggest that the increased SAM area in *OsbZIP47KD* seedlings is attributed to an increase in cell size and number. To understand some molecular corelates for SAM phenotypes, transcript levels for few known regulators of SAM size homeostasis were tested (Figure 1G) using SAM tissues from 25 DAG seedlings. The downregulation of *FCP1* and *FON2* (rice homologs of *CLV3*), *APO1* (*UFO1* homolog), *CYP734A4*, and *YUCCA6* was observed. Also, *CUC1*, the lateral meristem boundary marker (Aida et al., 1997; Takeda et al., 2011), showed a marginal reduction in expression. The downregulation of rice *CLV3* homologs in *OsbZIP47KD* SAM may contribute to the overall enlarged SAM size. Further, the increased cell size in L1 layer and its underlying cells could be attributed to a reduction in *CYP734A4* expression in SAM of *OsbZIP47KD* plants. Of note is the report that SAM cells in *CYP734A* RNAi plants are more vacuolated as compared to the WT which was suggested to indicate premature cell differentiation (Tsuda et al., 2014). Altogether, these gene expression effects of *OsbZIP47* can be related to abnormal SAM size homeostasis on its KD with novel effects on the components in the CLV-WUS pathway, on other meristem regulators, AUX, and BR phytohormone pathways.

### Late Heading Date and Altered Panicle Architecture of *OsbZIP47* Knockdown Plants

**FIGURE 2:**
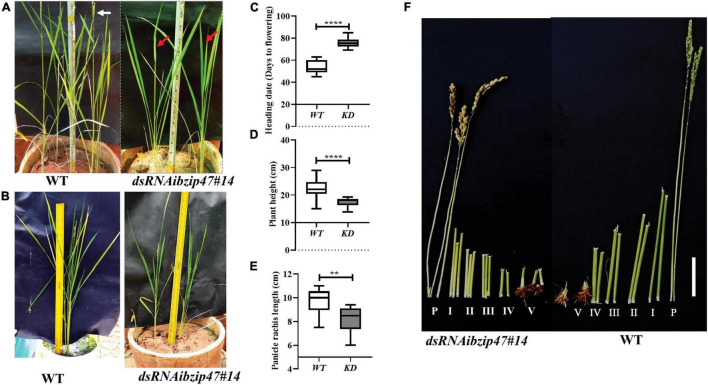
Phenotypic effects of *OsbZIP47KD* on plant growth and floral transition. **(A)** Flowering timing (SAM to inflorescence meristem (IM)/panicle meristem transition) in *OsbZIP47KD* plants is delayed by approximately 22 days as compared to the WT. White arrow indicates the booted panicle in WT and red arrow points to the absence of the booted panicle in a *OsbZIP47KD* plant of the same age. **(B)** Height of mature flowering plants shows that as compared to the WT, *OsbZIP47KD* plants are shorter. Quantitation of heading dates **(C)**, plant height **(D)**, and panicle rachis length **(E)** in WT and *OsbZIP47KD* plants. Data are shown as the mean ± s.d. (Student’s *t*-tests, **P < 0.01, ****P < 0.0001, *n* = 10). **(F)** Internodes in mature flowering WT and *OsbZIP47KD* plants are displayed and are numbered from I to V from the apical end. P is the panicle bearing node and internode. Bar, 2.5 cm. Shorter internodes from I to V in *OsbZIP47KD* contributes to the reduced height.

**FIGURE 3:**
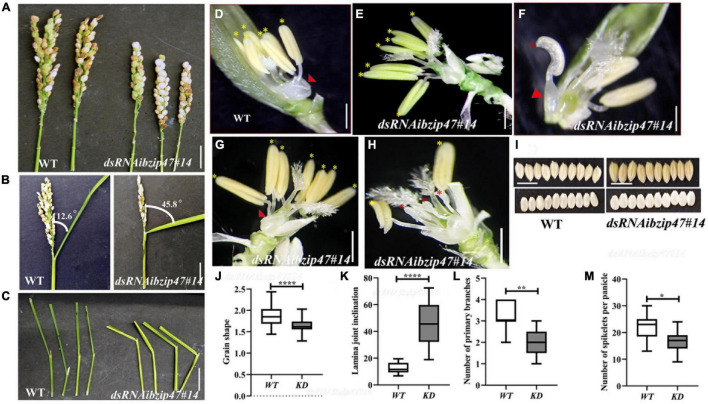
Floret organ numbers and organ development in *OsbZIP47KD* plants. **(A)** Shorter panicle *OsbZIP47KD* with reduced number of primary branches and spikelet number per panicle as compared to the WT plant. **(B,C)** Flag leaf angle in fully headed panicles shows increased lamina joint angle in *OsbZIP47KD* plant as compared to the WT. **(D)** WT floret organs shown after removal of lemma and the spikelet sterile lemmas. Red arrow points to the pair of lodicules and yellow asterisks to the six normal stamens. **(E–H)** Organ phenotypes in *OsbZIP4*7*KD* florets. **(E)** Floret with seven stamens (yellow asterisks) with normal filaments and anthers. **(F)** Floret with mildly deformed lodicule (red arrowhead), two of its six normal stamens (others were removed) and a chimeric second whorl organ with lodicule and stamenoid features (red asterisk). **(G)** Floret with deformed lodicule and seven normal stamens (yellow asterisk). **(H)** Floret with slightly deformed lodicule, short stamens, and shrunken anthers (red asterisk), two near normal stamens (yellow asterisk). **(I,J)** Grain morphology in *OsbZIP47KD* and WT plants. The length/width (L/W) ratio of *OsbZIP47KD* seeds was lower than that of WT seeds, suggesting increased grain width. Scale bar = 1 cm in panels **(A–I)**. **(J–M)** Statistical analysis (mean ± s. d.) of grain shape, lamina joint angle, primary branch number, and number of spikelets per panicles. Data in panels **(J)** [*n* = 40 in panel **(J)**], **(K)** (*n* = 10), **(L)** (*n* = 11), **(M)** (*n* = 9), Student’s *t*-tests, *P < 0.05, **P < 0.01, ***P < 0.001.

*OsbZIP47KD* plants are delayed by 20 days for SAM to IM transition. At this stage, *OsbZIP47KD* plant height was reduced compared to WT (Figures 2A–D) suggesting that in the WT, *OsbZIP47* promotes developmental transition from the vegetative to reproductive phase. The shorter plant height was due to poor stem internode elongation in the KD transgenics without change in internode number (Figure 2F). The panicle of KD plants showed developmental abnormalities, i.e., reduced inflorescence axis (panicle rachis) length (Figure 2E), reduced number of primary branches, and spikelets (Figures 3A,L,M and Supplementary Table 1). Together, these phenotypes indicate early progression of primary branch meristems to spikelets in *OsbZIP47KD* plants and point to a possible role of *OsbZIP47* in the temporal control of branch meristem indeterminacy. Moreover, *OsbZIP47KD* plants had greater flag leaf lamina joint angle as compared to the WT (Figures 3B,C,K). KD plants of *CYP734A4* (Tsuda et al., 2014), a gene with reduced expression in *OsbZIP47KD* plants (Figure 1G), also share this phenotype and have abnormal meristems. Plant architecture, flowering time, leaf angle, and inflorescence architectures all impact yield, grain shape, and size (Harder and Prusinkiewicz, 2013; Sakuma and Schnurbusch, 2020). Interestingly, seeds from *OsbZIP47KD* plants were altered for the length/width (L/W) ratio as compared to WT (Figures 3I,J), suggesting *OsbZIP47* impedes cell proliferation in the grain width direction, which is supported by a recent finding of Hao et al. (2021). In later sections of our study on *dsRNAiOsbZIP47* lines, we identify some molecular links underlying cell proliferation in the developing grain. We propose that the role of *OsbZIP47* in restriction of cell proliferation is likely attributed to the positive regulation of *EL2*, encoding a plant cyclin-dependent kinase inhibitor, and negative regulation of some key cell-cycle regulators i.e., *CYCLIN-D7-1* (*CYCD7;1*) and *MITOGEN-ACTIVATED PROTEIN KINASE KINASE 10* (*MKK10-1*) in WT panicles (Supplementary Dataset 1). Rice EL2 cell cycle inhibitory functions are proposed to link cell cycle progression with biotic and abiotic stress responses (Peres et al., 2007). In Arabidopsis, *AtCYCD7* expression is transcriptionally regulated by cell type-specific transcription factors that confine its expression to appropriate developmental window as ectopic expression triggered division (Weimer et al., 2018). The OsMKK10-1 paralog, OsMKK10-2 phosphorylates OsMPK6 *in vivo* (Ma et al., 2017). This is interesting since mutations of *OsMPK6* impair differentiation of L1 layer cells during early embryogenesis (Yi et al., 2016).

### *OsbZIP47* Contributes to Second and Third Whorls, Lodicule, and Stamen Organ Development

*OsbZIP47KD* floret phenotypes were largely restricted to lodicules and stamens (Figures 3D–H). The organ defects were grouped into four classes. Class I, representing 40% of *OsbZIP4KD* florets, had mild deformed lodicule (distal elongation) with normal stamen number (Supplementary Figure 3). In class II (∼28%), mild lodicule elongation occurred with abnormal short stamens and poorly developed anthers (Figure 3H). Florets of class III (∼20%) had partially deformed lodicules with an increase in stamen number to 7 (Figures 3E,G). In class IV (∼12%) florets had mildly elongated lodicules and chimeric organs with lodicule and stamen characteristics (Figure 3F). Also, in most florets of all classes, the lodicules were abnormally fused with lemma making dissection of the lemma from the floret difficult. Altogether, these data suggest that *OsbZIP47* contributes to floral organ development in the second and third whorls. In a complementary analysis, we examined consequences of ubiquitous overexpression of full-length *OsbZIP47*cDNA in transgenic rice. Surprisingly, none of the *OsbZIP47OX* lines had any notable phenotypic changes from the WT despite ∼10-fold overexpression in *OsbZIP47OX* panicle tissues (Supplementary Figure 4). A speculation is that *OsbZIP47* functions may depend on partners or that some post-translational modifications (PTMs) may modulate its functions, as was concluded from overexpression studies of Arabidopsis *AtPAN* (Chuang et al., 1999).

### Tissue Expression Profile of *OsbZIP47* Through Development

**FIGURE 4:**
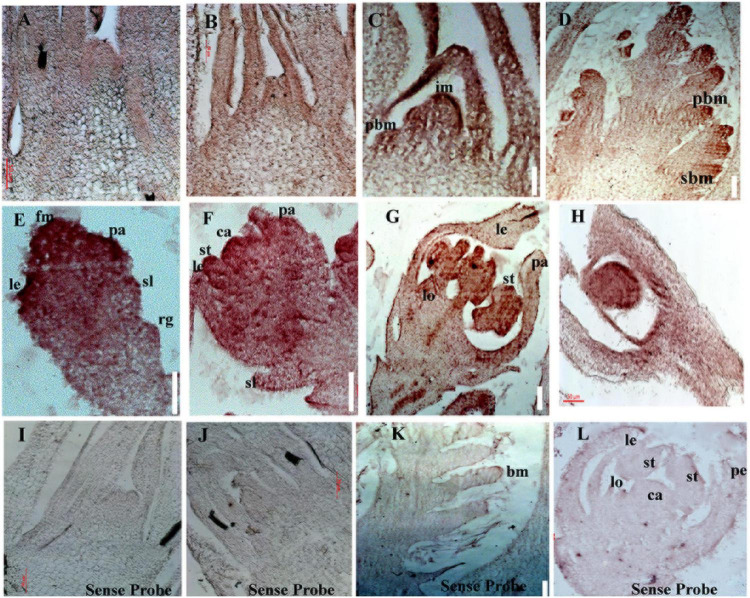
Spatial distribution of *OsbZIP47* transcripts in meristems and florets. **(A,B)** *OsbZIP47 in situ* RNA hybridization signal in SAM tissues from 5 DAG and 25 seedlings. Expression in SAM and in emerging leaf primordia. **(C,D)** Inflorescence meristem (IM) with emerging primary branch meristems show expression of *OsbZIP47* in the elongation meristem and primary branch meristems (prb), secondary branch meristems (srb), and in young leaves. **(E)** *OsbZIP47* transcripts in very early floret meristem (FM) with uniform spatial distribution of signal. **(F)** Floret uniform signal in the well-formed stamens (st), lemma/palea (le and pa) organ primordia, and in the central early carpel primordia. **(G)** High level of *OsbZIP47* expression in the lodicule and in stamens, and lower signal in the near mature lemma and palea. **(H)** *OsbZIP47* transcripts in the ovary wall and ovule. **(I,J)** SAM in 5 DAG and 25 DAG plants probe with sense probe as a negative control. **(K,L)** IM and near mature floret, respectively probed with sense RNA.

RNA *in situ* hybridization was performed to examine spatial distribution of *OsbZIP47* mRNA in various above ground meristems. These experiments confirmed transcripts in meristems that is consistent with the phenotypes of *OsbZIP47KD* plants. In SAM of wild type young seedlings (5 DAG and 25 DAG), transcripts were evenly distributed (Figures 4A,B). This pattern is somewhat different from maize *FEA4* where the signals are excluded from SAM stem cell niche and from incipient P0 leaf primordium (Pautler et al., 2015). During reproductive development, high levels of *OsbZIP47* transcripts are shown at the apical end of growing IM/rachis and at the ends of branch meristem (PBM and SBM, Figures 4C,D) which may relate to the poorly branched inflorescence of knockdown plants. In elongating primary and secondary branches (Figure 4D), transcript signal is mild and spatially uniform. In spikelet meristem (SM, Sp2, Figure 4E), and in floral meristems (Sp4-Sp6, Figure 4F), the signal is high and spatially uniform. However, in mature florets, *OsbZIP47* RNA was confined to the lodicule, stamen and carpel organ primordia, and differentiating organs (Figure 4G). Additionally, hybridization signal in carpel wall (c) and ovule (o) was observed (Figure 4H). Arabidopsis *pan* mutant flowers occasionally have multiple carpels up to three with deviated gynoecium (Running and Meyerowitz, 1996). We speculate *OsbZIP47* may have a minor role in carpel development or could be functionally redundant with floral C-class function genes. Thus, *OsbZIP47* is expressed in various above-ground meristems reflecting its diverse roles in different meristems.

### Heterodimerization of OsbZIP47 With Other Floral Meristem Regulators

**FIGURE 5:**
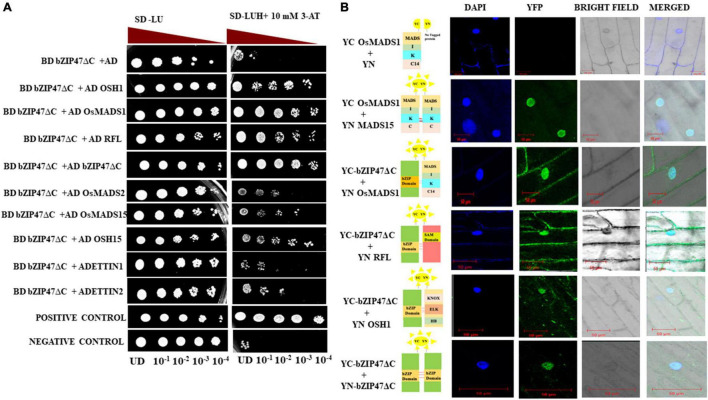
Interaction of OsbZIP47 with other meristem factors. **(A)** Yeast two hybrid (Y2H) assays with OsbZIP47ΔC protein (lacking 186–380 amino acids in C terminal) and predicted protein partners, such as OsMADS1 (MIKC14 domain), RFL, OsETTIN1, OsETTIN2, OSH1, OsMADS2, OsMADS15, and OSH15. Serial dilutions of yeast cells (PJ694A) spotted on media lacking histidine and supplemented with 10 mM 3-AT. Growth after five days at 30°C is shown. Yeast transformants with OsMADS15 in pGBDUC1 vector and OsMADS1 MIKC14 in pGADC1vector served as positive control for protein interaction. A combination of pGADC1 and pGBDUC1 empty vectors (without protein fusion) was the negative control. **(B)** Validation of Y2H protein interactions by Bimolecular Fluorescence Complementation (BiFC) assays in onion epidermal cells. YFP fluorescence was detected when OsbZIP47ΔC-cYFP fusion protein was co-expressed with OsMADS1-nYFP, RFL-nYFP, and OSH1 nYFP. Absence of YFP fluorescence in the negative control i.e., OsbZIP47ΔCYC and YN domain alone was the negative control. Bars = 50 μm.

Heterodimerization of transcription factors can modulate genome wide gene expression by modifying specificity and affinity to target DNA binding sites, and by integrating independent pathways controlled by two or more factors. Interacting partners of OsbZIP47 or of its Arabidopsis and Maize homologs are largely unknown. To understand molecular mechanism of OsbZIP47, we investigated its interaction with different transcription factors. Among the co-occurring motifs in genome-wide loci bound by OsMADS1, the motif for bZIP factor binding is enriched (Khanday et al., 2016). This was the basis for our hypothesis that OsMADS1 and members of OsbZIP family could function in complexes in early floral meristems. Further, the temporal co-expression profiles of *OsMADS1* and *OsbZIP47* overlap in developing panicles (Arora et al., 2007; Nijhawan et al., 2008); hence we tested interaction among these proteins using the heterologous yeast two hybrid assay (Figure 5). Additionally, to investigate possibility of OsbZIP47 heterodimerization with other meristem regulators, we relied on reports from genetic studies in Arabidopsis, maize and rice to curate and choose candidates for interaction assays (Das et al., 2009; Khanday et al., 2013; Deshpande et al., 2015; Pautler et al., 2015). OSH1, OsH15, ETTIN1, ETTIN2, and RFL emerged as candidates. We re-visited reports on the panicle and floret expression patterns of these meristem regulators to deduce if spatial co-expression of *OsbZIP47*, *OSH1* and *OSH15* could occur. RNA *in situ* patterns of *OSH1* in rice panicles and florets (Komatsu et al., 2001; Chu et al., 2006; Hu et al., 2015) and *OsbZIP47* transcript spatial profile (Figure 4) point to an overlap of *OsbZIP47* and *OSH1* transcripts in primary and secondary branch primordia and in a broad range of developing spikelet/floret meristems (Sp2–Sp8) (Supplementary Figure 5). Further, mutant *osh1* (Tsuda et al., 2011, 2014) and *OsbZIP47KD* plants share common phenotypes such as increased leaf lamina joint angle, short panicle with reduced number of spikelets and deformed stamens. Moreover, Pautler et al. (2015) reported that several gene loci are cobound by ZmKN1 (the ortholog of rice OSH1) and ZmFEA4 (ortholog of *OsbZIP47*). These findings together indicate likelihood of OsbZIP47 and OSH1 interactions for co-regulation of target genes. Also, OsH15, a closely related paralog of OSH1 (Tsuda et al., 2011), co-expresses with *OsbZIP47* in spikelet meristem/floret meristem stage 6 (Yoon et al., 2017; Supplementary Figure 5). As *OsbZIP47* KD caused abnormal floral phenotypes, it was intriguing to determine if OsbZIP47 could heterodimerize with OsH15. In Arabidopsis, *pan ettin* phenotypes suggest *AtPAN* and *AtETTIN/AUXIN-RESPONSIVE FACTOR3* (*ARF3*) redundantly regulate floral organ numbers and patterns (Sessions et al., 1997). Additionally, rice *ETTIN1* and *ETTIN2* RNAi lines have aberrant plant height, compromised panicle branching, and defects in stamen and carpel development (Khanday et al., 2013). Some of these phenotypes resembled those observed in *OsbZIP47KD* plants. Similarly, for mutants in *RFL*, the rice *AtLEAFY* ortholog, the alleles *apo2* and *ssc*, or the *RNAi RFL*KD (Kyozuka et al., 1998; Rao et al., 2008; Wang et al., 2017) plants share some phenotypes with *OsbZIP47KD* plants. The common phenotypes include shorter plant height, delayed flowering, reduced panicle rachis length, and branch complexity. Thus, we hypothesized that OsETTIN1, OsETTIN2, and RFL may interact with OsbZIP47 to modulate aspects of organ development. Based on these meta-analyses, protein partnership between OsbZIP47 and OSH1, OsH15, ETTIN1, ETTIN2, and RFL was tested by the Y2H assay. Moreover, we also tested the interactions of OsbZIP47 with OsMADS15 (an A Class APETALA1/FRUITFULL AP1/FUL-clade transcription factor) and OsMADS2 (a B class PISTILLATA/GLOBOSA-like protein). OsMADS15 regulates vegetative to reproductive floral transition and functions in specifying meristem identity (Kater et al., 2006; Kobayashi et al., 2012). OsMADS2 functions in partnership with OsMADS16/SUPERWOMAN1 (SPW) as a B-class complex (Lombardo et al., 2017; Kong et al., 2019). The delayed flowering phenotypes of *OsbZIP47KD* transgenics and the defects in the second and third whorl floral organs, justified our choice of OsMADS15 and OsMADS2, respectively. Since full length OsbZIP47 exhibited transcriptional transactivation activity in yeast (Supplementary Figure 6A), a C-terminal truncated version (OsZIP47ΔC) lacking 186-385 amino acids including the transcription activation domain was taken as bait protein in fusion with Gal4 BD. Prey proteins (OsMADS1, OSH1, OsH15, ETTIN1, ETTIN2, RFL, OsMADS15, or OsMADS2) were fused to GAL4 AD. The GAL4AD-OsMADS15 interaction with GAL4DB-OsMADS1 was taken as the positive control (Lim et al., 2000). In addition, homodimerization capability of OsbZIP47ΔC was tested. Growth pattern of transformed yeast cells on reporter media SD/-Leu-Ura-His + 10 mM3AT and the X-gal quantitative assays (Supplementary Figure 6B) suggested that OsbZIP47 can heterodimerize with OsMADS1, OSH1, and RFL. We also found a strong homodimerization of OsbZIP47ΔC protein (Figures 5A,B). Both OsMADS15 and OsH15 showed weak interactions with OsbZIP47 (Figure 5A), while ETTIN1, ETTIN2, and OsMADS2 showed no interaction. Further, we performed *in-planta* BiFC assays to substantiate the protein–protein interactions screened in Y2H assay. *OsbZIP47ΔC* was cloned upstream to the coding sequence of C-terminal region of split YFP to express *OsbZIP47ΔC*-cYFP fusion protein. The coding sequences of *OsMADS1*, *OsbZIP47ΔC*, *RFL, OsETTIN2*, and *OSH1* were cloned in frame downstream to the coding sequence of the N-terminal split YFP (nYFP) to express nYFP fusion proteins. These six different combinations of nYFP and cYFP fusion proteins were transiently co-expressed in onion epidermal cells. Nuclear YFP fluorescence signals confirmed protein interaction of OsbZIP47 with OsMADS1, OSH1, and RFL (Figure 5B). Thus, we suggest that OsbZIP47 partnership with OsMADS1, OSH1, and RFL could contribute to meristem functions, inflorescence complexity, and floret development.

### Transcriptome of Developing Inflorescences of *OsbZIP47* Knockdown Lines

**FIGURE 6:**
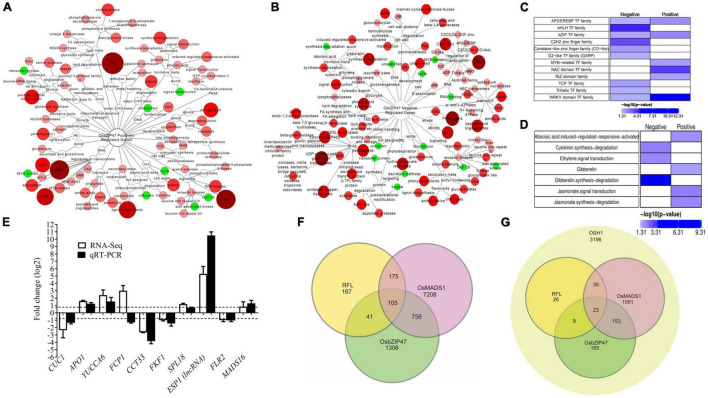
Differentially expressed genes (DEGs) and GO enrichment analysis of pathways downstream to OsbZIP47. Enrichment networks depicting pathways regulated by OsbZIP47. **(A)** positively regulated, and **(B)** negatively regulated pathways. The red and green color nodes represent enriched and depleted functional categories, respectively (*P* < 0.05). The size and color intensity of the node correlates with the over-representation of genes within the given class. **(C)** Enrichment map of transcription factor (TF) genes shows those encoding members from bHLH, G2-like, C2H2 zinc finger, TCP, Trihelix are underrepresented, whereas TFs, such as AP2/EREBP, MYB-related, NAC domain, TAZ, WRKY, and bZIP were overrepresented. **(D)** The 2,210 DEGs annotated in MSU-TIGR v7 were taken for GO-enrichment analysis for phytohormone metabolism and signaling. Cytokinin (CK) degradation, ethylene signal transduction genes, and gibberellin (GA)-synthesis-degradation were enriched in negatively regulated dataset, whereas, abscisic acid (ABA), GA pathway, and jasmonate (JA) pathways predominate in the positively regulated set. **(E)** RT-qPCR analysis of the relative fold-change for transcripts from several candidate target genes of OsZIP47 in 0.1 to 0.5 cm panicles from *OsbZIP47KD* as compared to WT tissues. The normalization of transcript level of each gene was done using *UBQ5* transcript levels. Error bars represent the standard deviation. **(F)** Venn diagram showing unique and overlapping sets of differentially expressed genes (DEGs) derived from *OsMADS1, OsbZIP47*, and *RFL* transcriptome datasets (Rao et al., 2008; Khanday et al., 2013; this study). **(G)** Genes implicated to be coregulated by *OsMADS1, OsbZIP47*, and *RFL* and bound by *OSH1* (Rao et al., 2008; Khanday et al., 2013; Tsuda et al., 2014; this study).

To capture gene expression landscape in *OsbZIP47KD* panicles, high throughput RNA-sequencing was carried out in two biological replicates of *OsbZIP47KD* and WT panicles (In2-In4, 1 mm to 5 mm panicles), and the DEGs (greater than two-fold change, *p*-value < 0.05) were extracted (Supplementary Dataset 1, Supplementary Materials and Methods). Further, DEGs were examined for gene ontology pathway enrichment. Among the DEGs, 1,945 genes were upregulated, and 855 genes were downregulated in *OsbZIP47KD* panicles (Supplementary Figure 7, Supplementary Dataset 1). Gene Ontology (GO) analysis of positively regulated gene set (Figures 6A,B and Supplementary Materials and Methods, Supplementary Dataset 2) revealed enrichment of RNA (regulation of transcription), lipid, CHO metabolism, signaling, development, and hormone metabolism pathways (Figure 6A). Whereas in the negatively regulated set, genes related to secondary metabolism, transport, cell wall, signaling, stress, hormone metabolism, and miscellaneous factors were overrepresented (Figure 6B). Not surprisingly, genes involved in hormone signaling and metabolism were controlled by *OsbZIP47*, both positively and negatively (Figures 6C,D). Specifically, jasmonate (JA) and abscisic acid (ABA) pathway genes are overrepresented in the positively regulated gene set. CK degradation and ethylene signal transduction genes are notable in the negatively regulated gene set. Interestingly, genes of GA pathway were enriched in the positively regulated gene set, whereas genes for GA-synthesis-degradation were enriched in the negatively regulated gene set (Figure 6D). Examples of *OsbZIP47* downstream genes that could interlink hormone pathways for panicle and floret development include *APETALA-2-LIKE TRANSCRIPTION FACTOR39* (OsAP2-39), 9- cis-epoxycarotenoid dioxygenase3 (*OsNCED3*), and *ELONGATED UPPER MOST INTERNODE1* (*OsEUI1*). *AP2-39* balances the antagonistic relation between ABA and GA by modulating the expression levels of *OsNCED3* and *OsEUI1* to regulate plant height, yield, and seed germination (Yaish et al., 2010; Shu et al., 2016). We observed positive regulation of *OsAP2-39* and *OsNCED3*, and negative regulation of *EUI1* by *OsbZIP47*. Thus, we suggest that *OsbZIP47* may enhance ABA biosynthesis and modulate GA biosynthesis possibly to regulate plant height and panicle rachis elongation (Figures 2D,E). Examples of other *OsbZIP47* downstream genes linked to different phenotypes in *OsbZIP47KD* plants are further discussed. Interestingly, in *OsbZIP47KD* inflorescences, whole transcriptome analyses showed higher transcript levels for the *CLV3* paralog genes, *FON2/4* and *FCP1.* Moreover, genes from the KNOX-WUS pathway, and isopentenyl-transferases, *IPT6*, and IPT8 that encode CK-biosynthesis rate limiting enzymes were also upregulated. These results together, suggest the roles of *OsbZIP47* in the regulation of panicle primary branch meristems and secondary branch meristems. Reduced transcript expression was observed for *SQUAMOSA PROMOTER BINDING PROTEIN-LIKE 7* (*SPL7*), *OsMADS16*, and *YABBY* domain factor-*DROOPING LEAF* (*DL*) in *OsbZIP47KD*. The *SPL7* regulates inflorescence meristem and spikelet transition (Dai et al., 2018). *OsMADS16* and *DL* regulate lodicule, stamen, and carpel development (Nagasawa et al., 2003; Yamaguchi et al., 2004). Moreover, the F-box gene, *APO1* with roles in spikelet and floret development was also deregulated (Supplementary Dataset 1 and Figure 6E; Ikeda et al., 2005, 2007). These findings corelate with the *OsbZIP47KD* inflorescence branching and floret organ defects (Figures 2, 3). The positive regulation of *CUC1* by *OsbZIP47* supports plausible mechanism for its influence on organ whorl boundaries (Takeda et al., 2011; Figure 6E and Supplementary Dataset 1) and could explain the development of chimeric floral organs in *OsbZIP47KD* transgenics. Transcription factors control the dynamics of hormone signaling pathways by modulating gene expression levels. Transcription factors genes that are deregulated in *OsbZIP47KD* panicles include bHLH gene members (22 genes), Co-like Zn finger (5 genes), TCP class 1 (2 genes), and TCP class 2 (1 gene), trihelix (4 genes), C2H2 zinc (20 genes) (Figure 6C and Supplementary Dataset 2). The genes for transcription factors that are positively regulated by *OsbZIP47* are from NAC class, WRKY class, and MYB-related class genes. Among the latter class is OsLHY (LATE ELONGATED HYPOCOTYL)/CCA1 (CIRCADIAN CLOCK ASSOCIATED1) which functions in photoperiodic flowering, plant tillering, and grain yield (Wang et al., 2020; Sun et al., 2021). We speculate that the delayed flowering phenotype of *OsbZIP47KD* plants can be associated with the positive regulation of *OsLHY* by *OsbZIP47*. We also speculate that positive regulation of *DWARF AND RUNTISH SPIKELET2/FERONIA like Receptor 2* (*DRUS2/FLR2*) may contribute to architecture, fertility, and seed yield (Li et al., 2016; Figure 6E and Supplementary Dataset 1). To obtain a predicted list of candidate direct genes and targets of OsbZIP47, we queried the dataset of deregulated genes in *OsbZIP47KD* panicles for the occurrence of *cis* motif typical to the TGA-subclade within the large family of bZIP factors in the rice, Arabidopsis, and maize genomes. Arabidopsis TGA sub-family includes AtPAN, the homolog of OsbZIP47, and AtPAN binds to the core *cis* regulatory element TGACG (Gutsche and Zachgo, 2016). Among the 2,210 differentially expressed annotated (MSU-TIGR v7) genes, a large number displayed the core motif TGACG in their TSS-promoter proximal regions (-500 bp to + 100 bp from TSS; Supplementary Dataset 3). Interestingly, the TGACG core motif occurred three times in this region of the *FCP1* locus hinting that *FCP1* deregulation is likely a direct effect of OsbZIP47. Other predicted direct targets that relate to developmental functions of OsbZIP47 are *APO1*, *GNP1* (*GA20Ox1*), *CYCD7*, *OsSPL7*, *OsIAA20*, and *GRX6*, to name a few. Other gene targets could be regulated by degenerate *cis* elements related to “core motif” or by “core motif” in other distal regions of these loci. This *in silico* prediction of downstream targets of OsbZIP47 provides an extra level of confidence to the transcriptome-based deregulated gene set and can facilitate DNA–protein interaction studies. Overall, these results give a snapshot of *OsbZIP47* molecular functions in inflorescence tissues and give leads to its unique *vs.* evolutionarily conserved roles for panicle meristem transitions and floral organ development.

### Comprehensive Datamining of Transcriptome Datasets of *OsMADS1*, *OsbZIP47*, *RFL*, and Genome Binding Dataset for *OSH1*

Extending our findings of OsbZIP47 interaction with OsMADS1, RFL, and OSH1, we carried out meta-analyses of published transcriptome datasets affected in mutants of these partner proteins. To identify candidate genes for co-regulation by these factors, the differential transcriptome in *dsRNAiOsbZIP47KD*, dsRNAi*OsMADS1*, and dsRNAi*RFL* panicles were examined (Rao et al., 2008; Khanday et al., 2013). First, we aligned genes from three different transcriptomic datasets for this meta-study (Supplementary Materials and Methods). The 2,210 (as annotated by MSU-TIGR v7) deregulated genes in *dsRNAiOsbZIP47KD* panicles were examined for overlap with 8,246 affected genes in *dsRNAiOsMADS1KD* transcriptome (Figure 6F and Supplementary Dataset 4; Khanday et al., 2013). Among 758 candidate genes co-regulated by both OsbZIP47 and OsMADS1 (Figure 6F and Supplementary Dataset 4), 204 genes were downregulated in both *OsbZIP47KD* and *OsMADS1KD* lines. These genes included Gibberellin-regulated protein precursor expressed (*GASR3*), auxin-responsive *SMALL AUXIN-UP RNA 11* (*OsSAUR11*), *DRUS2/FLR2*, and *JASMONATE ZIM-DOMAIN12* (*TIFY11D/OsJAZ12*) (ZIM domain transcription factor). Another group of genes (386 out of 758 genes) were upregulated in both *OsbZIP47KD* and *OsMADS1KD* panicle datasets. This sub-set includes *OsMADS16*, *GNP1* (*GRAIN NUMBER PER PANICLE1*) and genes that regulate hormone signaling, such as *OsIAA20, YUCCA7, PROBABLE AUXIN EFFLUX CARRIER COMPONENT 5B* (*PIN5B)*, and *ETHYLENE INSENSITIVE LIKE 4* (*EIL*). Among the 758 candidate genes co-regulated by OsbZIP47 and OsMADS1, a subgroup of 153 genes (Figure 6G) are also bound by OSH1 (Tsuda et al., 2014). Striking among this sub-set are: *IAA20*, *YUCCA7, SPLIT-HULL* (*SPH*), and *DRUS2*/*FLR2*. Similarly, 41 genes (Figure 6F) are common to the DEGs in *OsbZIP47KD* panicles (RNA-Seq) and a low-density microarray study of panicles from dsRNAi*RFL* KD plants (Rao et al., 2008). Interestingly, 32 out of 41 genes were downregulated in both these datasets including ethylene signaling gene, *ACO1* which regulates internode elongation (Iwamoto et al., 2011), JA signaling gene, *TIFY11D* (Kim et al., 2009), and the *EMBRYOSAC1* (*OsEMSA1*) involved in embryo sac development (Zhu Q. et al., 2017). Finally, we identified 105 DEGs common to the differential transcriptome in *RFLKD*, *OsMADS1KD*, and *OsbZIP47KD* panicles (Figure 6F). The notable genes include *ETHYLENE INSENSITIVE-LIKE GENE 2* (*EIL2*), *ALLENE OXIDE SYNTHASE* (*AOS1*), and *GIBBERELLIN 2-OXIDASE 9* (*GA2OX9*). A subset of 23 genes are potentially regulated by OSH1 (Figure 6G; Tsuda et al., 2014). We infer that these transcription regulators possibly multimerize in one or more forms of complexes to regulate meristem development in rice. Overall, these findings hint that protein complexes, plausibly heterogeneous, with the combinations of OsbZIP47 and its varied partner factors may spatially and temporally co-ordinate downstream gene expression during panicle development, spikelet, and floret development.

### Redox-Dependent DNA Binding of OsbZIP47

**FIGURE 7:**
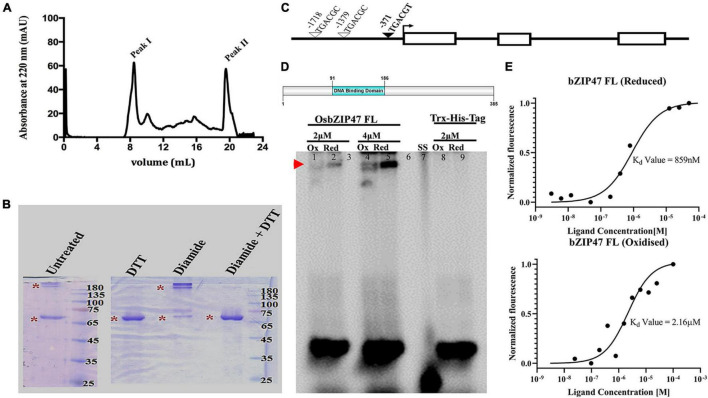
OsbZIP47 oligomerization and the effects of its redox status on DNA binding. **(A)** Size-exclusion chromatography (SEC) profile for OsbZIP47 protein/oligomer on a Superdex 200 increase column. **(B)** Mobility of purified OsbZIP47-Trx protein on non-reducing 10% of SDS-PAGE after treatment with 2 mM of diamide (an oxidizing agent), or with 20 mM DTT (a reducing agent), or with 2 mM diamide followed by 20 mM DTT. **(C)** Schematic representation of the *FCP1* gene locus. Exons are open white boxes, introns are shown as black lines, and the predicted *cis* motifs for bZIP DNA binding domain (DBD) are represented as inverted triangles. The DNA binding properties of OsbZIP47FL protein was tested for the TGACGT *cis* motif located at -371bp of *FCP1* (marked with a filled inverted triangle). **(D)** Schematic representation of OsbZIP47 FL protein (amino acid 1 to 385) used for EMSA and microscale thermophoresis assays. EMSA assays with full-length OsbZIP47 (62 Kda protein) shows retarded mobility of DNA-protein complex for the OsFCP1 locus (as indicated by red arrow). Lanes 1 and 2 correspond to the oxidized and reduced form of bZIP47 FL protein incubated with 2 μM of oligo. Lanes 4 and 5 represent the oxidized and reduced form of the same protein, incubated with 4 μM of oligo. Lanes 8 and 9 correspond to Trx-Tag protein incubated with 4 μM of probe. Lanes 3 and 6 are blank. Unbound dsDNA oligonucleotide is at the bottom. **(E)** Microscale thermophoresis-based assay to determine the dissociation constant for OsbZIP47 full-length and OsFCP1 dsDNA oligo interaction. 80 nM of fluorescently labeled protein was taken for binding with DNA sequences from OsFCP1 locus taken at a wide concentration range (100 μM to 3.05 nM). The OsbZIP47 full length protein was reduced with 20 mM DTT or oxidized with 1mM of oxidized glutathione. OsbZIP47 full length protein shows increased binding in the reduced state as compared to the oxidized state.

DNA binding by Arabidopsis AtPAN is redox-sensitive due to the five Cysteine residues in the extended N-terminal domain (Gutsche and Zachgo, 2016; Supplementary Figure 8). Unlike AtPAN homologs from diverse species, rice OsbZIP47 lacks this domain. All proteins share a conserved Cys in the C terminal transcription transactivation domain (AtPAN Cys340/OsbZIP47 Cys269). Cys17 in OsbZIP47 is conserved in monocot species, while Cys196 is unique to *OsbZIP47.* Thus, though the rice and *Arabidopsis* proteins are homologs, they differ in size and in the overall number of cysteine residues. To examine OsbZIP47 oligomerization and the effects of its redox status on binding to target gene *cis* DNA elements, the full length (FL) OsbZIP47 protein was expressed in bacteria. The DNA binding activity of AtPAN is regulated by *S*-glutathionylation of the conserved Cys340 by AtROXY1, a glutaredoxin redox enzyme (Li et al., 2009; Gutsche and Zachgo, 2016). The corresponding conserved Cys 269 in OsbZIP47 may also render the rice protein to be redox-sensitive for biochemical activity. To determine if OsbZIP47 FL protein forms higher order self-oligomers, SEC with the purified Trx-His OsbZIP47 (62 Kda) protein was done and the elution of the protein in the column void volume (Figure 7A) suggested either aggregation or the formation of high order oligomers in the given condition. To examine the latter possibility, the purified OsbZIP47-His-Trx protein was treated with 2 mM of diamide, an oxidizing agent. In parallel, another aliquot of the protein was treated with 20 mM DTT, a reducing agent, and both treated protein fractions were analyzed on a non-reducing SDS-PAGE gel. The oxidized OsbZIP47-His-Trx sample had slower mobility whereas the reduced OsbZIP47-His-Trx sample migrated with the expected mobility for a ∼68 Kda protein. Importantly, we found that the effects of the oxidizing agent (diamide) can be reversed by DTT treatment. These data show that OsbZIP47 oligomerization is affected by its redox status (Figure 7B). Recently, OsbZIP47 maize ortholog, FEA4 was shown to switch its oligomerization status following redox change (Yang et al., 2021). Next, we tested the DNA binding affinity of OsbZIP47 to the TGACGT *cis* motif (predicted for OsbZIP47 DNA binding) present at around -371 bp upstream of the start codon in the *OsFCP1* locus (Figure 7C). The latter is a downstream gene target whose expression levels are affected in the SAM and in the panicles of *OsbZIP47KD* plants. The OsbZIP47 FL protein status was altered by incubation with the reducing agent DTT (20 mM) or with the oxidant diamide (2 mM) for 30 min prior to the incubation with the TGACGT motif containing DNA substrate. OsbZIP47 FL protein bound to the TGACGT motif under reducing conditions. Interestingly, incubation with diamide decreased OsbZIP47 FL-DNA interaction. Thus, electrophoretic mobility shift assays showed redox-sensitive DNA-binding of the OsbZIP47 FL protein (Figure 7D). Further, we quantified the binding affinity of OsbZIP47 FL with *cis* element from the *OsFCP1* locus using microscale thermophoresis (MST). To this end, OsbZIP47 FL protein was labeled with the RED-NHS 2nd Generation Dye (MO-L011, Nanotemper GmbH) and was mixed with increasing concentrations of *OsFCP1* oligos until saturation. The fluorescent signals obtained with increasing ligand concentrations followed a clear sigmoidal binding curve. As expected, OsbZIP47 FL protein displayed a stronger binding affinity to the *OsFCP1* locus in its reduced state, with a dissociation constant K_*d*_ of 815 nM, as compared to K*_*d*_* of 2.16 uM in the oxidized state (Figure 7E). The differential K_*d*_ values confirm a redox sensitive DNA–protein interaction. The Trx-His tag protein was taken as a negative control in these assays and no interaction between protein and ligand was detected (Supplementary Figure 9). Overall, the results of our qualitative and quantitative data suggest that affinity of *OsbZIP*47 FL binding to DNA was redox-dependent despite the absence of the extended Cys rich N-terminal domain commonly found in homologs from other species. Recently, OsbZIP47 close ortholog, ZmFEA4 was shown to interact with three GRX proteins to modulate its redox status and DNA accessibility (Yang et al., 2021).

## Discussion

Arabidopsis PAN, maize FEA4, and rice bZIP47 proteins share a significant degree of similarity throughout their lengths with the most conserved region being the DNA binding domain (Supplementary Figure 8; Chuang et al., 1999; Nijhawan et al., 2008). Species-specific developmental roles for these factors may therefore arise from interacting co-regulators, from protein modification of these transcriptional regulators, and through variations in their downstream genes and pathways. Arabidopsis *AtPAN* has pleiotropic vegetative and reproductive growth effects. Flowers in the *pan* mutant are characterized by an increase in the floral organ number without a corresponding increase in FM size (Running and Meyerowitz, 1996; Chuang et al., 1999). Another study reported early flowering of *pan* mutant plants in long-day and short-day grown plants having an enlarged SAM and inflorescence meristems (Maier et al., 2011). Maize mutant *zmfea4* have enlarged vegetative SAMs, severely fasciated inflorescences, and florets with reduced stamen numbers (Pautler et al., 2015). We show that *OsbZIP47KD* plants have abnormalities in the shoot meristem size homeostasis similar to the enlarged SAM of maize *fea4* mutant. Yet, other phenotypes are unique to rice *OsbZIP47KD* lines, for instance, delayed flowering, increased stamen numbers, chimeric floral organs, and subtle changes to grain size and shape. Hence, despite redundancy of bZIP transcription factors, the phenotypes of single mutants, such as *atpan1*, *zmfea4*, and *OsbZIP47KD* indicate OsbZIP47 has evolutionarily conserved as well as unique roles in the vegetative and reproductive development. The partnership of OsbZIP47, with meristem regulators, OsMADS1, RFL, and OSH1 (KNOX1/STM), its oligomeric and redox status could relate to its functions in different meristems. This partnership and the findings that emerge from the differential transcriptome in *OsbZIP47KD* panicles allowed us to map OsbZIP47-regulated downstream genes, and those potentially dependent on its co-regulators.

### Meristem Development in Vegetative and Reproductive Phase

The enlarged SAM in *OsbZIP47KD* seedlings is superficially similar to that of maize *fea4*, yet there are underlying subtle differences in rice KD plants. A detailed phenotyping of SAMs from *OsbZIP47KD* seedlings showed increased cell size of L1 layer and its underlying cells suggesting precocious cell differentiation. Further, SAMs of *OsbZIP47KD* showed increased transcript signal of cell proliferation marker, H4 as indicated by *in situ* hybridization. The downregulation of *FON2*, *FCP1*, *YUCCA6, CUC1, APO1*, and *CYP734A4* in SAM, suggests several complex pathways by which OsbZIP47 contributes to SAM size and plant growth by modulating cell proliferation and differentiation. Notable here is the regulation of *CYP734A*, which as a direct target of OSH1 is suggested to repress premature cell differentiation in meristems (Tsuda et al., 2014). This observation together with our data on protein interactions between OsbZIP47 and OSH1 supports a plausible mechanism by which OsbZIP47-OSH1 partnership could regulate *CYP734A* expression with ensuing effects on meristem and lateral primordia development. Further, phytohormones, CK, AUX, and GA are also essential for cell division and organ differentiation (Leibfried et al., 2005; Zhao et al., 2010; Su et al., 2011). Transcriptome profiling of *OsbZIP47KD* panicles shows deregulated expression levels for different phytohormone related genes including *KNOTTED1-LIKE11*, *IPT1*, *IPT8*, *OsGA3OX2 OsGA2OX3*, *OsGA2OX4*, Os*GA20OX1*, *YUCCA6*, and *YUCCA7.* Plant meristems are under redox control by reactive oxygen species (ROS), the by-products of aerobic metabolism. ROS levels control the expression of *WUS* and the activity of TCP class1 transcription factors to balance cell proliferation and differentiation in Arabidopsis SAM (Viola et al., 2013; Zeng et al., 2017). Like TCP class1, proteins containing cysteines with low pKa values are sensitive to cellular redox status (Martins et al., 2018). Glutaredoxins (GRX) reduce cellular ROS level and interact with different proteins including AtPAN1, ZmFEA4, and OsbZIP47 to modulate their activity by modifying their redox state (Li et al., 2009; Laporte et al., 2012; Schippers et al., 2016; Hao et al., 2021; Yang et al., 2021). Arabidopsis *pan* and maize *fea4* mutants exhibited increased meristem size (Maier et al., 2011; Pautler et al., 2015). Contrastingly, single, double, and triple mutations of different GRX paralogs in maize showed progressive reduction of meristem size, proposing a model where GRXs balance redox status and activity of ZmFEA4 to control meristem size (Yang et al., 2021). Interestingly, in transcriptome profiling of *OsbZIP47KD* panicles, *OsGRX6* was downregulated whereas *OsGRX16* and *OsGRX20* were upregulated (Supplementary Dataset 1), suggesting a mechanism by which *OsbZIP47* could be regulating meristem development. We propose OsbZIP47 functions as an integrator of the WUS-CLV and KNOX pathways for meristem development as it regulates the expression levels of key signaling factors in both pathways. Also, genes predicted to have roles in floral organ primordia differentiation such as *OsBLH1* (*BEL1*-like homeodomain), *OsKANADI1*, and *CUC1* are deregulated in *OsbZIP47KD* panicle tissues. Interestingly, reproductive panicle branching phenotypes of *OsbZIP47KD* panicles resemble *apo1* and *apo2/rfl* mutants (Ikeda et al., 2005, 2007; Rao et al., 2008; Ikeda-Kawakatsu et al., 2009; Deshpande et al., 2015). Thus, the interactions between RFL and OsbZIP47 could positively regulate the panicle meristem branch identity and its developmental transitions. One example of a target gene for co-regulation by OsbZIP47 and RFL is *CUC1*. Further, the elevated transcript levels of *APO1* in *OsbZIP47KD* panicle tissues hints that *OsbZIP47* in the WT panicle suppresses the expression of *APO1* which we speculate may affect the partnership with *APO2/RFL.* From this, we anticipate that *OsbZIP47* could have evolved to regulate some unique molecular pathways for vegetative and reproductive phase meristem growth and development.

### Transition of Shoot Apical Meristem to Inflorescence Meristem

Knockdown of rice *OsbZIP47* showed delayed flowering (Figure 2A). This trait is common in mutants or KD transgenics in *OsMADS1* and *APO2/RFL* that encode OsbZIP47 protein partners (Jeon et al., 2000; Rao et al., 2008; Ikeda-Kawakatsu et al., 2012; Kannan et al., 2021). These observations support our hypothesis that these factors function in “one or more” complexes. Panicle transcript analysis in KD transgenics indicates that *OsbZIP47* can promote flowering by fine-tuning the expression of several flowering time regulators that are upstream to florigens, *HEADING DATE* 3a (*Hd3a*) and *RICE FLOWERING LOCUS* (*RFT*), and by controlling the expression of circadian clock-associated genes. Examples of genes from these two categories are *O. sativa LATE FLOWERING* (*OsLF*), *LATERAL ORGAN BOUNDARY DOMAIN* (*OsLBD38*), *INDETERMINATE DOMAIN 6* (*OsIDD6*), *FLAVIN-BINDING, KELCH REPEAT F-BOX1* (*OsFKF1*), *PHYTOCLOCK 1*(*OsPCL1*), and *OsLHY*/*CCA1*. Several rice flowering time quantitative trait loci (QTLs) also influence grain traits (Chen et al., 2014; Zhu Y.J. et al., 2017; Ma et al., 2019). The effect of *OsbZIP47KD* on rice grain shape is not reported for maize *fea* kernels suggesting unique effects of *OsbZIP47* on grain size and shape in rice as also reported by Hao et al. (2021). Our transcriptomic analysis identified a set of grain shape genes regulated by *OsbZIP47.* Examples of this category are *LONG GRAIN 3* (*OsLG3*), *GRAIN SHAPE GENE ON CHROMOSOME 9* (*GS9*), *GRAIN WIDTH QTL on chromosome 7* (*GW*) and *FLOURY ENDOSPERM 2* (*FLO2*). *GS9 positively* controls the grain size by altering the cell division along with BR signaling (Zhao et al., 2018). Interestingly, we noted increased expression of *CYCD7;1* in *OsbZIP47KD* panicles (Supplementary Dataset 1). This is remarkable as in Arabidopsis, the tissue and stage-specific control of this G1-S phase cell cycle gene controls the cell division in different contexts, with ectopic expression driving increased cell division and expansion in the embryo and the endosperm (Collins et al., 2012; Weimer et al., 2018). With this, we postulate that *OsbZIP47* links flowering time, cell cycle, and BR signaling to regulate grain shape.

### Regulation of Inner Floral Organ Identity and Specification

Consistent with *OsbZIP47* expression in the second and third whorl organs of near mature florets (Sp6–Sp8), we observed lodicule and stamen differentiation defects in *dsRNAiOsbZIP47* florets. Increased stamen numbers, with degenerated anthers on short filaments and lodicule-stamen chimeric organs support roles for OsbZIP47 in organ differentiation. Interestingly, in *OsbZIP47KD* florets, the higher transcript abundance of *OsMADS16* (homolog of *AtAP3*) and *DL*, a contributor to Class C function in rice florets (Supplementary Dataset 2), are indicative of some distinct effects in rice florets. Overexpression of *OsMADS16* can increase stamen numbers and form stamenoid carpels without any effects on lodicules (Lee et al., 2003). More recently, rice transgenics with a modified repressive *OsMADS16* (OsMADS16-SDX repressor domain fusion) exhibited indehiscent anthers (Sato et al., 2012). These phenotypes are akin to third whorl organ differentiation defects seen in *OsbZIP47KD* florets. Microsporogenesis in anthers of Arabidopsis flowers requires *SPOROCYTELESS/NOZZLE* (*SPL/NZZ*), a target of Class B and C organ identity factors (Ito et al., 2004). In line with this, we noted upregulation of *OsSPL*, possibly an effect of increased *OsMADS16* in *OsbZIP47KD* florets. Since we did not detect OsbZIP47 interaction with OsMADS2 in the Y2H assay, we speculate that OsbZIP47 regulates stamen differentiation by modulating the expression of *OsMADS16.* Upstream regulators of *OsMADS16* are *OsDL* and *FON2* (Ikeda et al., 2007; Xu et al., 2017). Both are upregulated in *OsbZIP47KD* inflorescence. The upregulated transcript of *FON2* in *OsbZIP47KD* inflorescence suggests that *OsbZIP47KD* stamen phenotypes are *OsMADS16*-mediated.

### Biochemical Properties of OsbZIP47 Can Underlie Its Unique Functions and Downstream Effects

Multiple sequence alignment shows that OsbZIP47 shares nearly 50% of amino acid identity with homologs across diverse species. A common feature among many bZIP47 proteins, except OsbZIP47 and Bamboo PH01000727G0540, is a variably extended N terminal domain (Supplementary Figure 8). DNA binding activity of Arabidopsis PAN is redox-sensitive due to the presence of five Cysteine (Cys) amino acids in the extended N-ter domain and the conserved C-ter Cys340 in the transcription transactivation domain (Gutsche and Zachgo, 2016). OsbZIP47 protein has only three Cys (Cys17, Cys196, and Cys269). Cys17 is represented in all the monocot species. Cyse269 (Cys 340 of AtPAN) is conserved in all homologs compared here (Supplementary Figure 8). Cys196 is unique to OsbZIP47. Interestingly, among proteins compared here, wheat TAE56722G002 has the maximum number of 11 Cys. These observations hint that the number of Cys residues in this clade of bZIP proteins may have evolved for species-specific roles, plausibly for the adoption of unique structures with effects on tissue-specific target gene expression. Despite being a shorter protein with fewer Cys residues, OsbZIP47 showed redox-dependent DNA binding to *OsFCP1*, a downstream gene whose expression was upregulated in *OsbZIP47KD* panicles. Yang et al. (2021) demonstrated that OsbZIP47 maize ortholog, FEA4 interacts with three GRX proteins to modulate its redox status and DNA accessibility proposing a model by which redox status of FEA4 mediate meristem size. In rice, OsGRX19 or MICROSPORELESS1 (OsMIL1) is a potential glutaredoxin redox enzyme for OsbZIP47 as it is a homolog of the glutaredoxin redox enzyme, AtROXY1 and ZmMSCA1 from Arabidopsis and maize, respectively (Timofejeva et al., 2013; Yang et al., 2015). Interaction between OsMIL1 and TGA1 in yeast and the reduction of glutathionylation of OsbZIP47 by ROXY homolog, WG1 are both established (Hong et al., 2012; Hao et al., 2021). Indehiscent anthers phenotype is common to *OsbZIP47KD* and *mil1* mutant (Hong et al., 2012) leading us to propose S-glutathionylation of OsbZIP47 could be important for the development of anther. Overall, we uncover conserved as well as unique functions and mechanisms of OsbZIP47 that support meristem growth and determinacy during vegetative and reproductive development leading to grain formation. Together, these functions make OsbZIP47 a potential locus for allele mining and crop improvement.

## Data Availability Statement

The datasets presented in this study can be found in online repositories. The names of the repository/repositories and accession number(s) can be found below: https://www.ncbi.nlm.nih.gov/geo/query/acc.cgi?acc=GSE196747.

## Author Contributions

UV, SP, RR, MZ, and OA designed the research. SP, RR, MZ, and OA performed research and experiments. RP performed bioinformatic analyses. Data analyses and manuscript preparation was done by SP, RR, MZ, RP, and UV. All authors read and approved the final manuscript.

## Conflict of Interest

The authors declare that the research was conducted in the absence of any commercial or financial relationships that could be construed as a potential conflict of interest.

## Publisher’s Note

All claims expressed in this article are solely those of the authors and do not necessarily represent those of their affiliated organizations, or those of the publisher, the editors and the reviewers. Any product that may be evaluated in this article, or claim that may be made by its manufacturer, is not guaranteed or endorsed by the publisher.

## Footnotes

1
https://rapdb.dna.affrc.go.jp/

2
http://rice.plantbiology.msu.edu/
