# Supplementary material for: OsbZIP47 Is an Integrator for Meristem Regulators During Rice Plant Growth and Development
Source: Front Plant Sci. 2022 Apr 13;13:865928. doi: 10.3389/fpls.2022.865928 (PMC9044032; doi:10.3389/fpls.2022.865928)
Supplement: Supplementary file 1 [file Presentation_1.pdf]

**SUPPLEMENTAL DATA**

**Supplemental Figure S1.** Schematic of T-DNA segment in the *pUbi:OsbZIP47*-RNAi construct and assessment of knockdown of *OsbZIP47* transcripts in transgenics.

**Supplemental Figure S2.** Spatial expression patterns of *H4*-Histone transcripts in 25-day old SAM of wild-type and *OsbZIP47KD* seedlings.

**Supplemental Figure S3**. Floral phenotypes of *OsbZIP47KD* plants.

**Supplemental Figure S4**. Generation of *OsbZIP47* overexpression lines and phenotype in T1 generation plants.

**Supplemental Figure S5.** Schematics showing comparative spatial co-expression of *OsbZIP47*, *OSH1* and *OSH15* in different developmental stages of rice floret.

**Supplemental Figure S6.** Yeast one hybrid assay to test transcriptional autoactivation of *HIS3* reporter by full length OsbZIP47 and quantitative β-galactosidase activity assay of the interacting protein pairs tested in yeast two hybrid assay.

**Supplemental Figure S7.** Volcano plot display of differentially expressed genes in panicles of *OsbZIP47KD versus* WT.

**Supplemental Figure S8.** Multiple Sequence Alignment of OsbZIP47 with its orthologues showing conserved and non-conserved residues.

**Supplemental Figure S9.** SDS PAGE gel showing purified proteins used for DNA protein interaction analysis and MST assay for binding of Trx-His tag to OsFCP1 annealed oligos.

**Supplemental Table S1.** Phenotypes of T3 generation *OsbZIP47* knockdown plants.

**Supplemental Table S2.** List of primers/oligonucleotides used in this study.

**Supplemental Data Set S1**. List of genes deregulated by 2-fold ($P < 0.05$) in panicles of *OsbZIP47KD* lines as compared to the wild-type transcriptome.

**Supplemental Data Set S2.** List of DEGs for Gene Ontology Enrichment Analysis.

**Supplemental Data Set S3.** List of genes deregulated in *OsbZIP47KD* panicles with TGA bZIP core motif in promoter regions.

**Supplemental Data Set S4.** List of genes co-regulated by OsbZIP47, OsMADS1 and RFL, bound or not bound by OSH1.

**Supplemental Figure S1**

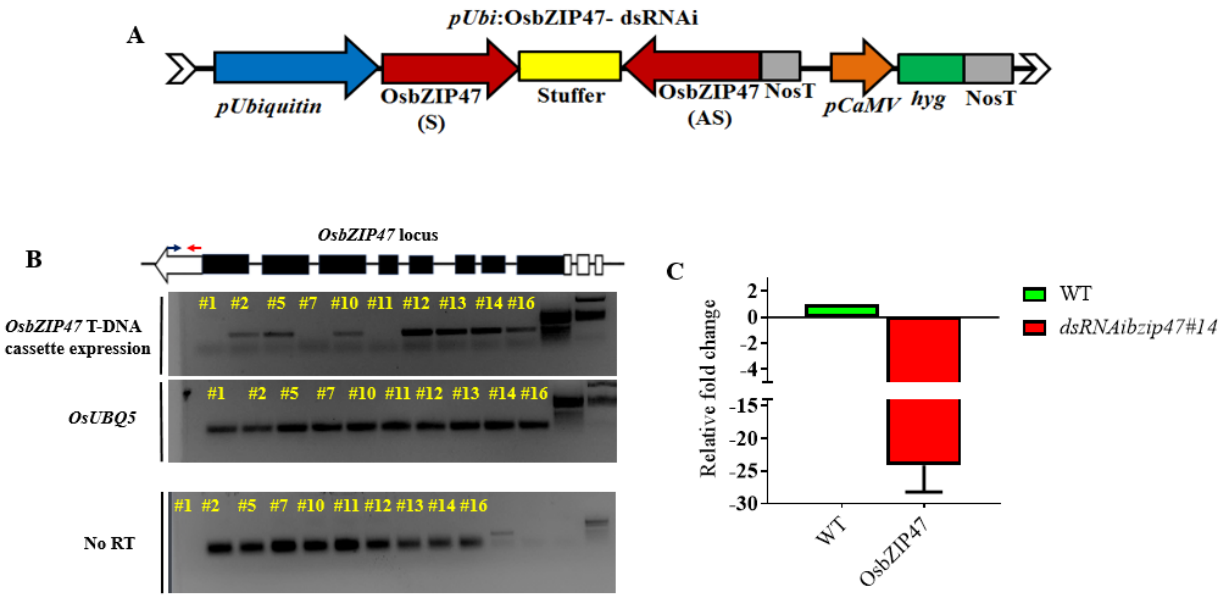


**Supplemental Figure S1. (A)** Schematic of T-DNA segment of *pUbi:OsbZIP47*-RNAi. **(B)** Estimation of transgenic cassette-based expression of shRNAs in different *OsbZIP47 KD* lines. RNA from leaves of each line was used for RT-PCR to detect the transgenic shRNA. Red and black arrows indicate the location of the primers used to amplify a 226bp DNA fragment from 3'UTR that would generate *OsbZIP47* hairpin RNA. Leaf tissue was chosen for these experiments as the endogenous *OsbZIP47* is not expressed in leaves. **(C)** Estimation, by qRT-PCR, of the level of knockdown of endogenous Os*bZIP47* expression in panicle tissues (0.1-0.5cm) from line number #14. The expression in wild type and in *OsbZIP47KD#14* was normalized to *Ubiquitin 5* internal control and the graph plotted after taking WT to 1.

**Supplemental Figure S2**

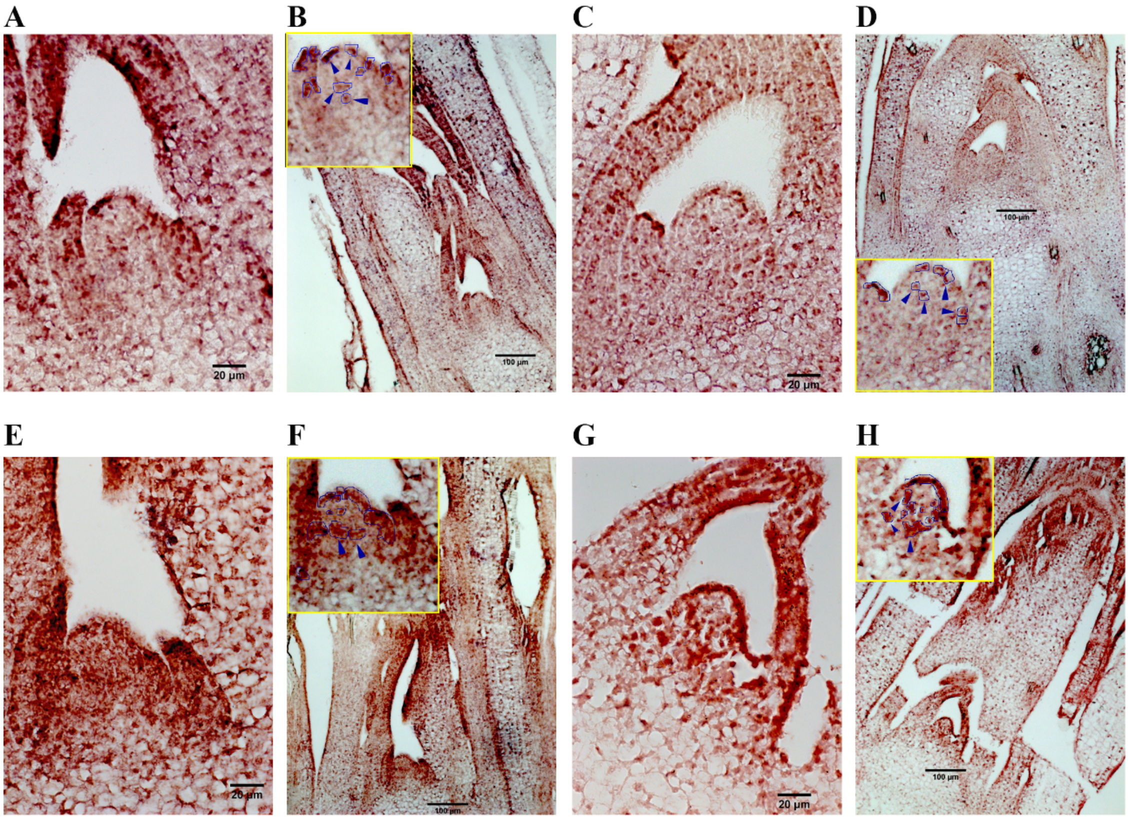


**Supplemental Figure S2.** RNA *in situ* localization of histone H4 transcripts in longitudinal sections of vegetative shoot apical meristem (SAM) of wild- type and *dsRNAibZIP47#14* in 25-day old seedlings. **(A-D)** wild type SAMs at 40X (A, C) and 10X (B, D) magnification. Blue arrowheads in the insets shown in B and D panels point at patches of cells (outlined with dashed blue line) with RNA hybridization signal. **(E-H)** *dsRNAibZIP47#14* SAMs at 40X (E, G) and 10X magnification (F, H). Blue arrowheads in the insets shown in F and H panels point at patches of cells (outlined with dashed blue line) with RNA hybridization signal. The number of cells

expressing histone H4 and the level of signal is increased in the median longitudinal sections of SAM in *dsRNAibZIP47#14* plants indicating altered cell division. Scale bars are of 20 µm in A, C, E, G and 100 µm in B, D, F, H.

**Supplemental Figure S3**

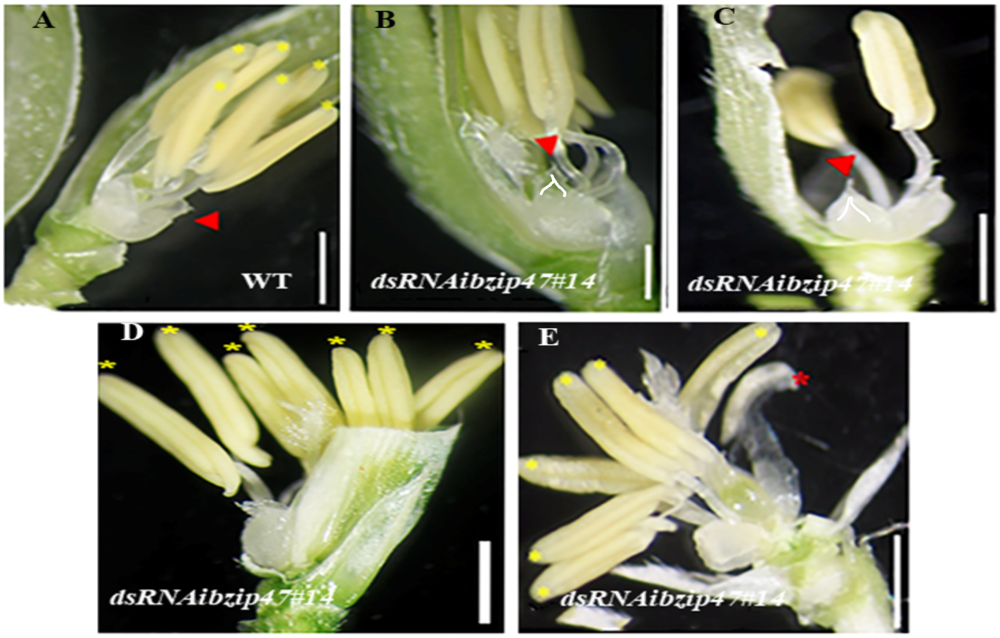


**Supplemental Figure S3.** Floral phenotypes of *OsbZIP47* knockdown plants. **(A)** A typical WT floret where lemma and sterile lemma were removed to expose and visualize inner floral organs. Red arrow indicates the pair of lodicules and yellow asterisks point to the six normal stamens. The central organ, carpel is not fully visible, due to lodicule and stamens. **(B-E)** Different floret from *OsbZIP47* KD lines, with floral organs phenotypic defects. **(B and C)** Mild distal elongation of the lodicule (outline marked with white line) with six normal stamens (stamens were dissected when needed to display the lodicule). **(D)** A floret with mildly deformed lodicule and with 7 apparently normal stamens (yellow asterisk). **(E)** A floret with a deformed lodicule, six stamens and a chimeric lodicule-stamenoid organ apparently from the second whorl (whorl position same as the normal lodicule).

**Supplemental Figure S4**

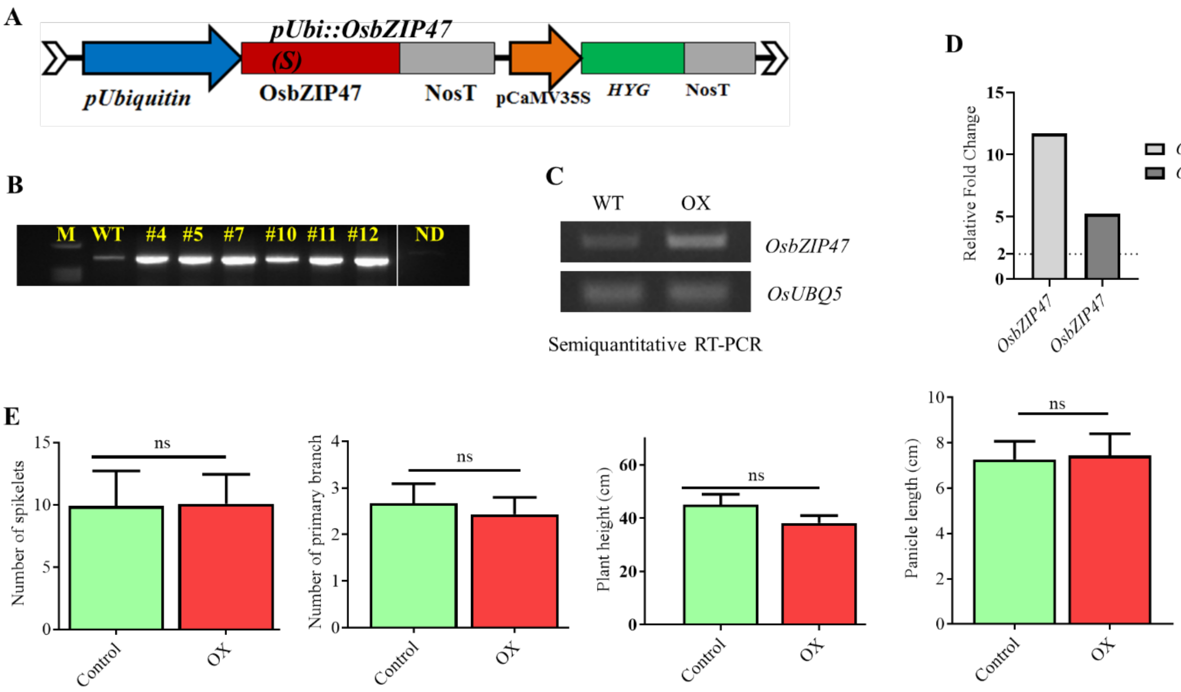


**Supplemental Figure S4.** Generation of *OsbZIP47* overexpression lines and phenotype in T1 generation plants. **(A)** Schematic representation of the T-DNA segment used to generate transgenics with *OsbZIP47* overexpression. The full-length cDNA of *OsbZIP47* was expressed from the maize ubiquitin promoter with NosT terminator at 3'end. **(B)** Validation of the transgenic status of different T0 lines by genomic DNA PCR for the selection marker Hygromycin. **(C and D)** Semi-quantitative RT-PCR and RT-qPCR based validation of overexpression of bZIP47 transcript in panicle tissues of transgenic line#4. Fold change values were determined by comparing the normalized expression levels in two biological replicates (light grey and dark grey bars) of *OXbZIP47* tissue to WT tissues. **(E)** Bar graphs show plant height, panicle length, number of primary branches on the panicle, and number of spikelets for line number #4 in the T1 generation. None of these traits in transgenic show any significant variation from the control vector transformed transgenic WT.

**Supplemental Figure S5**

**A**

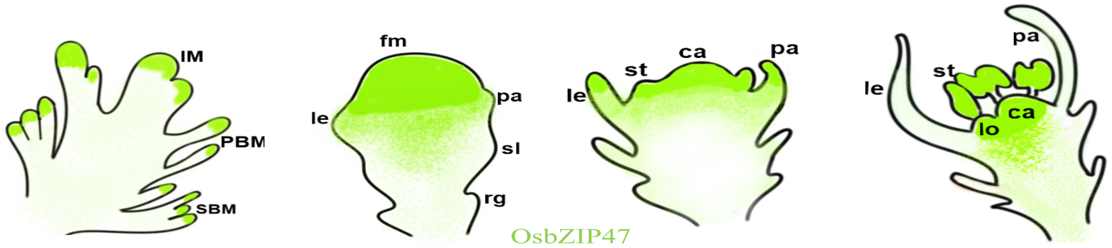


**B**

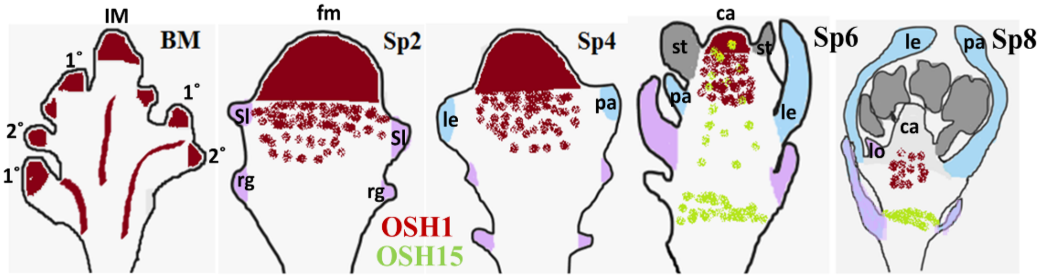


**Supplemental Figure S5.** Schematic representation of the comparative spatial co-expression analysis of *OsbZIP47* with meristem regulators, i.e., *OSH1* and *OSH15* deduced from the data in Fig. 4 and public datasets. **(A)** Spatial localization of *OsbZIP47* transcripts by RNA *in situ* hybridizations in WT inflorescence meristem (IM), branch meristems (PBM, SBM), spikelet meristem (SM), floral meristem/FM (Sp2), floral meristem (Sp4-Sp6) and mature florets (Sp8) tissues show strong ubiquitous expression in all meristems. Expression in mature florets is restricted to inner organs, lodicule, stamen and carpel. **(B)** Schematic representation for the expression of *OSH1* and *OsH15* transcripts from publicly available data (Chu et al., 2006; Hu et al., 2015; Komatsu et al., 2001; Yoon et al., 2017). This suggests that the expression of *OSH1* transcripts (in reddish-brown filled circles/ stipples) overlaps with the expression of *OsbZIP47* (panel A light green shading) in Branch meristem/BM, Spikelet meristem/SM (Sp2), floral meristem/FM (Sp4-Sp6) staged tissue whereas the expression of *OSH15* (light green circles/ stipples in panel B) partially overlaps with *OsbZIP47* (panel A light green shading) in floral meristem/FM (Sp6) staged tissue.

**Supplemental Figure S6**

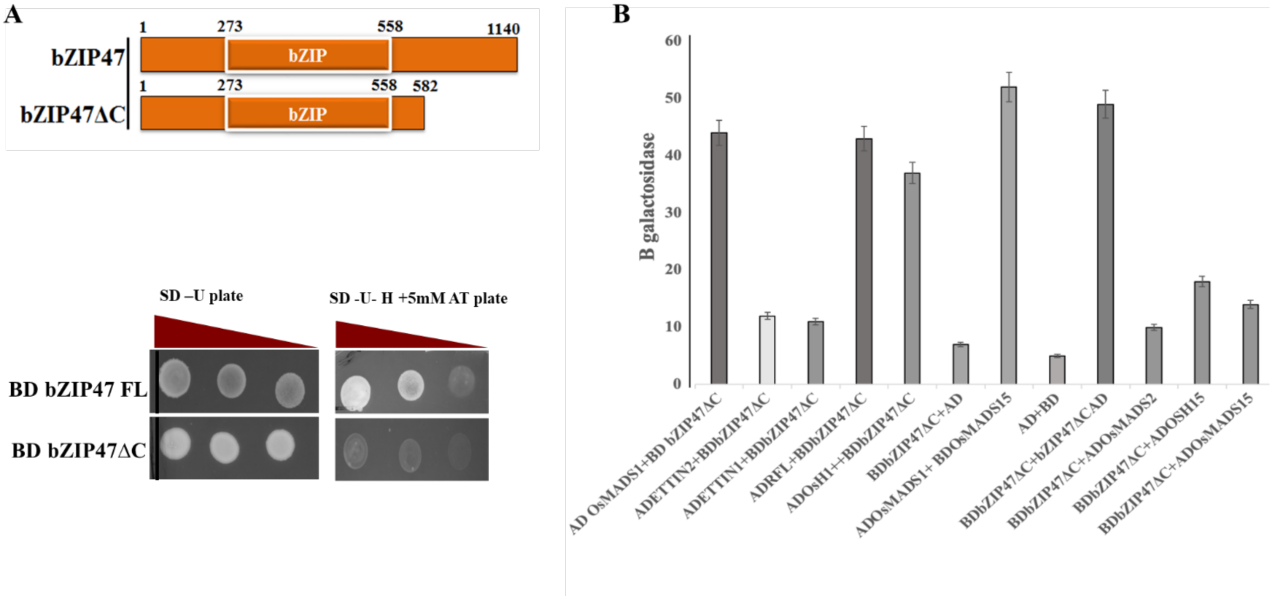


**Supplemental Figure S6**. **(A)** Top panel: Schematic representation of full-length and truncated OsbZIP47 proteins used for testing transactivation by yeast one-hybrid. Bottom left panel shows growth of yeast transformants selected on SD-Ura media. Bottom right panel shows growth of yeast transformants with only pGBDUC1 OsbZIP47 FL on His reporter plate (SD/-Ura - His +3AT) which was compared to transformants with only pGBDUC1OsbZIP47ΔC (deleted for transactivation domain). Growth in the top row of colonies indicates the full length protein can carry out transcriptional transactivation of *HIS3* reporter to confer growth in the absence of Histidine and in presence of a competitive inhibitor 3AT (3-Amino-1,2,4-triazole). **(B)** Quantitative β-galactosidase activity assay of the indicated protein interactions tested in pairs. ONPG was used as a substrate for detection of the *lacZ* reporter gene expressed enzyme and absorbance was determined at 420 nm. Yeast transformants with OsMADS15 expressed from pGBDUC1 vector and OsMADS1 MIKC14 from the pGADC1 vector served as the positive control for protein interaction. A combination of pGADC1 and pGBDUC1 empty vectors (without protein fusions) was the negative control.

**Supplemental Figure S7**

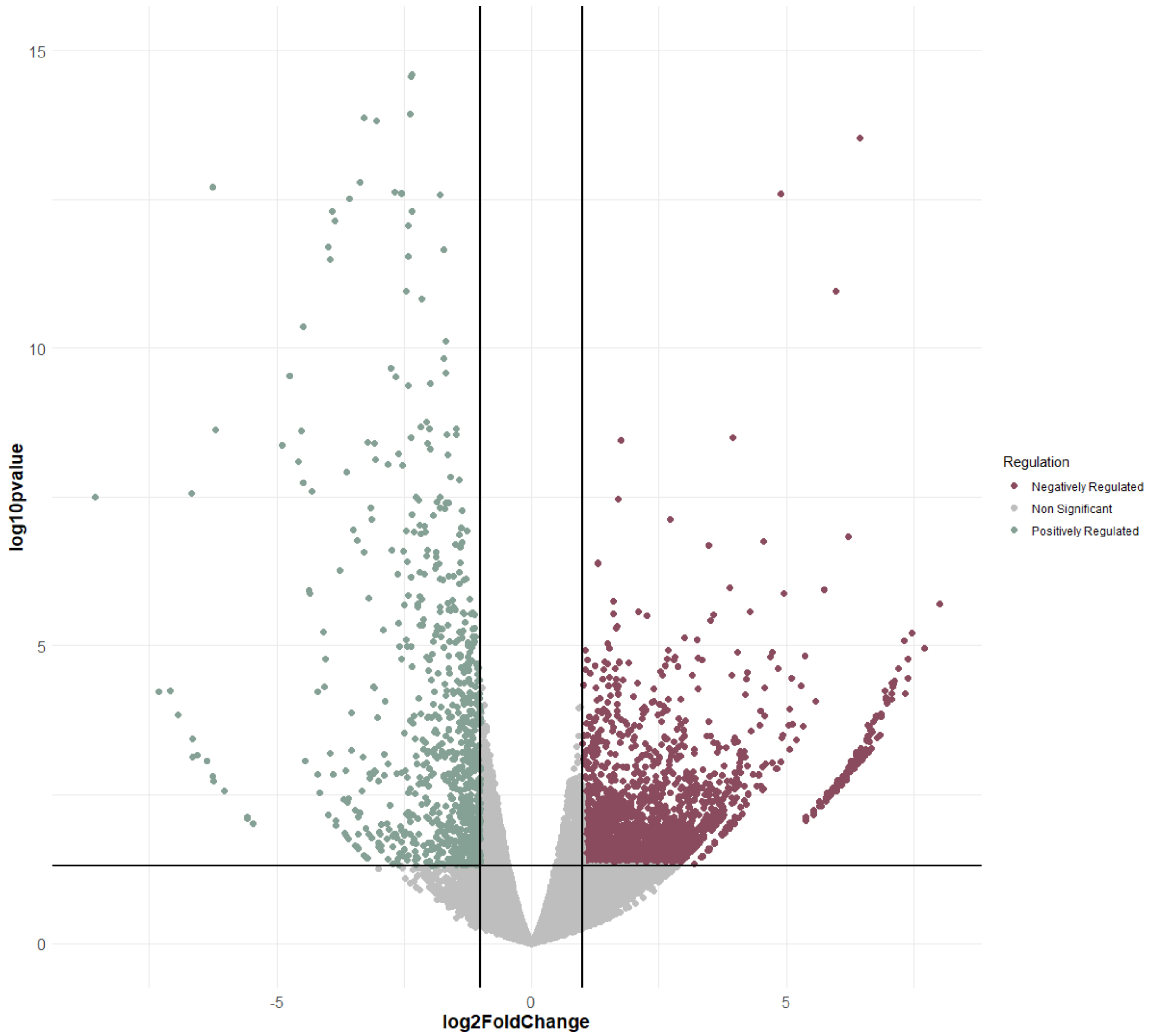


| Differentially expressed genes (DEGs) in *dsRNAibZIP47* vs WT from RNA-sequencing. DEG with absolute $\log_2$ fold change ≥ 1, p-value ≤ 0.05 | | | |
|---|---|---|---|
| **Tested genes** | Significantly DEG's | Upregulated genes | Downregulated genes |
| **29532** | 2800 | 1945 | 855 |

**Supplemental Figure S7.** Volcano plot display of differentially expressed genes in panicles of dsRNAi *OsbZIP47* transgenics as compared to WT tissues. Cyan dots represent genes/transcripts of positively regulated genes in WT panicles and maroon dots represents genes/transcripts of

negatively regulated genes in WT panicles. Grey dots represents genes/transcripts with non-significant deregulation.

**Supplemental Figure S8**

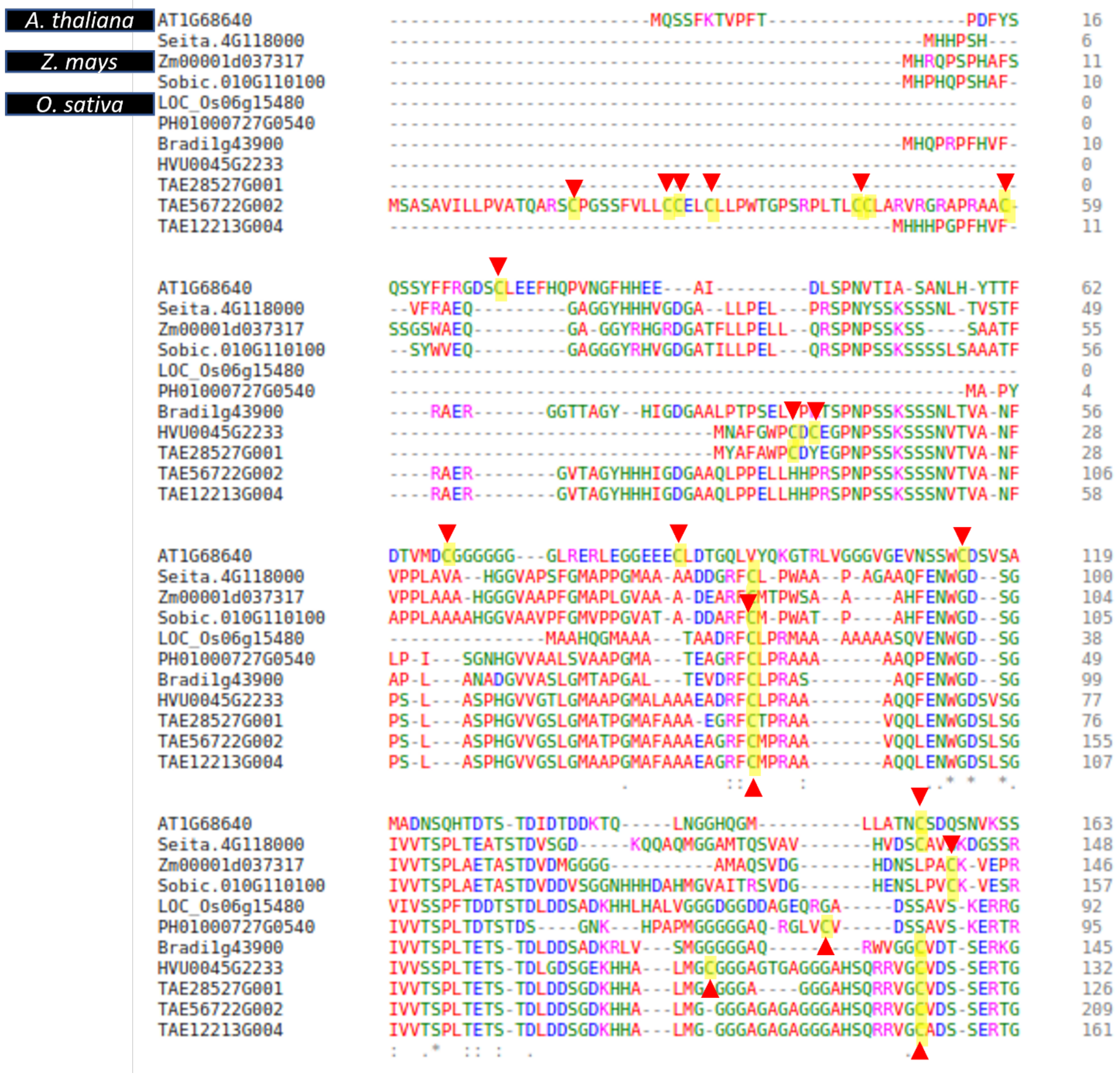

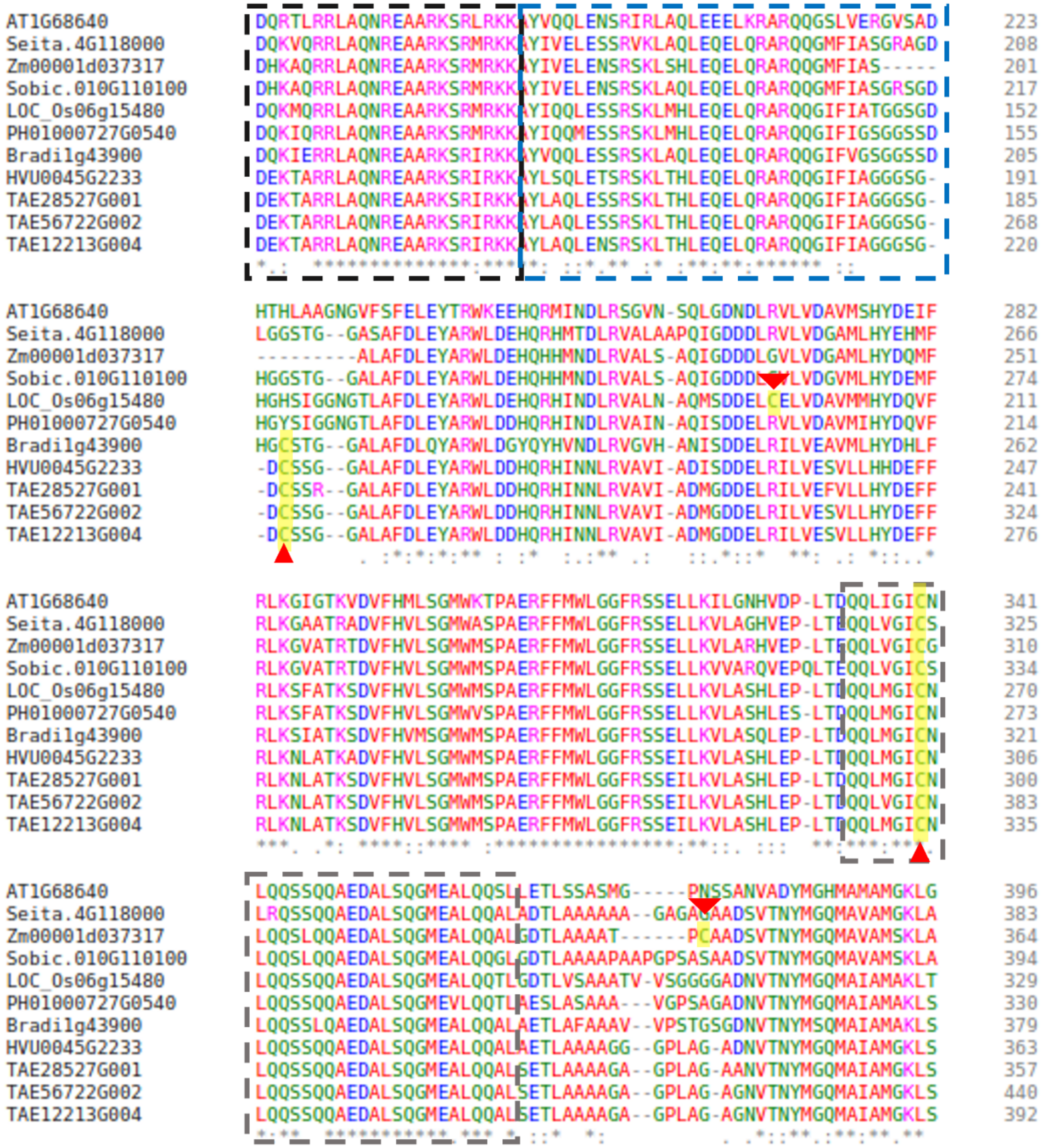
AT1G68640 DQRTLRRLAQNREAARKSRLRKKAYVQQLENSRIRLAQLEEELKRARQQGSLVERGVSAD 223
Seita.4G118000 DQKVQRRLAQNREAARKSRMRKKAYIVELESSRVKLAQLEQELQRARQQGMFIASGRAGD 208
Zm00001d037317 DHKAQRRLAQNREAARKSRMRKKAYIVELENSRSKLSHLEQELQRARQQGMFIAS----- 201
Sobic.010G110100 DHKAQRRLAQNREAARKSRMRKKAYIVELENSRSKLAQLEQELQRARQQGMFIASGRSGD 217
LOC_Os06g15480 DQKMQRRLAQNREAARKSRMRKKAYIQQLESSRSKLMHLEQELQRARQQGIFIATGGSGD 152
PH01000727G0540 DQKIQRRLAQNREAARKSRMRKKAYIQQMESSRSKLMHLEQELQRARQQGIFIGSGGSSD 155
Bradi1g43900 DQKIERRLAQNREAARKSRIRKKAYVQQLESSRSKLAQLEQELQRARQQGIFVGSGGSSD 205
HVU0045G2233 DEKTARRLAQNREAARKSRIRKKAYLSQLETSRSKLTHLEQELQRARQQGIFIAGGGSG- 191
TAE28527G001 DEKTARRLAQNREAARKSRIRKKAYLAQLESSRSKLTHLEQELQRARQQGIFIAGGGSG- 185
TAE56722G002 DEKTARRLAQNREAARKSRIRKKAYLAQLESSRSKLTHLEQELQRARQQGIFIAGGGSG- 268
TAE12213G004 DEKTARRLAQNREAARKSRIRKKAYLAQLENSRSKLTHLEQELQRARQQGIFIAGGGSG- 220
AT1G68640 HTHLAAGNGVFSFELEYTRWKEEHQRMINDLRSGVN-SQLGDNDLRVLVDAVMSHYDEIF 282
Seita.4G118000 LGGSTG--GASAFDLEYARWLDEHQRHMTDLRVALAAPQIGDDDLRVLVDGAMLHYEHMF 266
Zm00001d037317 ---------ALAFDLEYARWLDEHQHHMNDLRVALS-AQIGDDDLGVLVDGAMLHYDQMF 251
Sobic.010G110100 HGGSTG--GALAFDLEYARWLDEHQHHMNDLRVALS-AQIGDDD VLVDGVMLHYDEMF 274
LOC_Os06g15480 HGHSIGGNGTLAFDLEYARWLDEHQRHINDLRVALN-AQMSDDELCELVDAVMMHYDQVF 211
PH01000727G0540 HGYSIGGNGTLAFDLEYARWLDDHQRHINDLRVAIN-AQISDDELRVLVDAVMIHYDQVF 214
Bradi1g43900 HGCSTG--GALAFDLQYARWLDGYQYHVNDLRVGVH-ANISDDELRILVEAVMLHYDHLF 262
HVU0045G2233 -DCSSG--GALAFDLEYARWLDDHQRHINNLRVAVI-ADISDDELRILVESVLLHHDEFF 247
TAE28527G001 -DCSSR--GALAFDLEYARWLDDHQRHINNLRVAVI-ADMGDDELRILVEFVLLHYDEFF 241
TAE56722G002 -DCSSG--GALAFDLEYARWLDDHQRHINNLRVAVI-ADMGDDELRILVESVLLHYDEFF 324
TAE12213G004 -DCSSG--GALAFDLEYARWLDDHQRHINNLRVAVI-ADMGDDELRILVESVLLHYDEFF 276
AT1G68640 RLKGIGTKVDVFHMLSGMWKTPAERFFMWLGGFRSSELLKILGNHVDP-LTDQQLIGICN 341
Seita.4G118000 RLKGAATRADVFHVLSGMWASPAERFFMWLGGFRSSELLKVLAGHVEP-LTEQQLVGICS 325
Zm00001d037317 RLKGVATRTDVFHVLSGMWMSPAERFFMWLGGFRSSELLKVLARHVEP-LTEQQLVGICG 310
Sobic.010G110100 RLKGVATRTDVFHVLSGMWMSPAERFFMWLGGFRSSELLKVVARQVEPQLTEQQLVGICS 334
LOC_Os06g15480 RLKSFATKSDVFHVLSGMWMSPAERFFMWLGGFRSSELLKVLASHLEP-LTDQQLMGICN 270
PH01000727G0540 RLKSFATKSDVFHVLSGMWVSPAERFFMWLGGFRSSELLKVLASHLES-LTDQQLMGICN 273
Bradi1g43900 RLKSIATKSDVFHVMSGMWMSPAERFFMWLGGFRSSELLKVLASQLEP-LTDQQLMGICN 321
HVU0045G2233 RLKNLATKADVFHVLSGMWMSPAERFFMWLGGFRSSEILKVLASHLEP-LTDQQLMGICN 306
TAE28527G001 RLKNLATKSDVFHVLSGMWMSPAERFFMWLGGFRSSEILKVLASHLEP-LTDQQLMGICN 300
TAE56722G002 RLKNLATKSDVFHVLSGMWMSPAERFFMWLGGFRSSEILKVLASHLEP-LTDQQLVGICN 383
TAE12213G004 RLKNLATKSDVFHVLSGMWMSPAERFFMWLGGFRSSEILKVLASHLEP-LTDQQLMGICN 335
AT1G68640 LQQSSQQAEDALSQGMEALQQSLLETLSSASMG-----PNSSANVADYMGHMAMAMGKLG 396
Seita.4G118000 LRQSSQQAEDALSQGMEALQQALADTLAAAAAA--GAGA AADSVTNYMGQMAVAMGKLA 383
Zm00001d037317 LQQSLQQAEDALSQGMEALQQALGDTLAAAAT------PCAADSVTNYMGQMAVAMSKLA 364
Sobic.010G110100 LQQSLQQAEDALSQGMEALQQGLGDTLAAAAPAAPGPSASAADSVTNYMGQMAVAMSKLA 394
LOC_Os06g15480 LQQSSQQAEDALSQGMEALQQTLGDTLVSAAATV-VSGGGGADNVTNYMGQMAIAMAKLT 329
PH01000727G0540 LQQSSQQAEDALSQGMEVLQQTLAESLASAAA---VGPSAGADNVTNYMGQMAIAMAKLS 330
Bradi1g43900 LQQSSLQAEDALSQGMEALQQALAETLAFAAAV--VPSTGSGDNVTNYMSQMAIAMAKLS 379
HVU0045G2233 LQQSSQQAEDALSQGMEALQQALAETLAAAAGG--GPLAG-ADNVTNYMGQMAIAMGKLS 363
TAE28527G001 LQQSSQQAEDALSQGMEALQQALSETLAAAAGA--GPLAG-AANVTNYMGQMAIAMGKLS 357
TAE56722G002 LQQSSQQAEDALSQGMEALQQALSETLAAAAGA--GPLAG-AGNVTNYMGQMAIAMGKLS 440
TAE12213G004 LQQSSQQAEDALSQGMEALQQALSETLAAAAGA--GPLAG-AGNVTNYMGQMAIAMGKLS 392

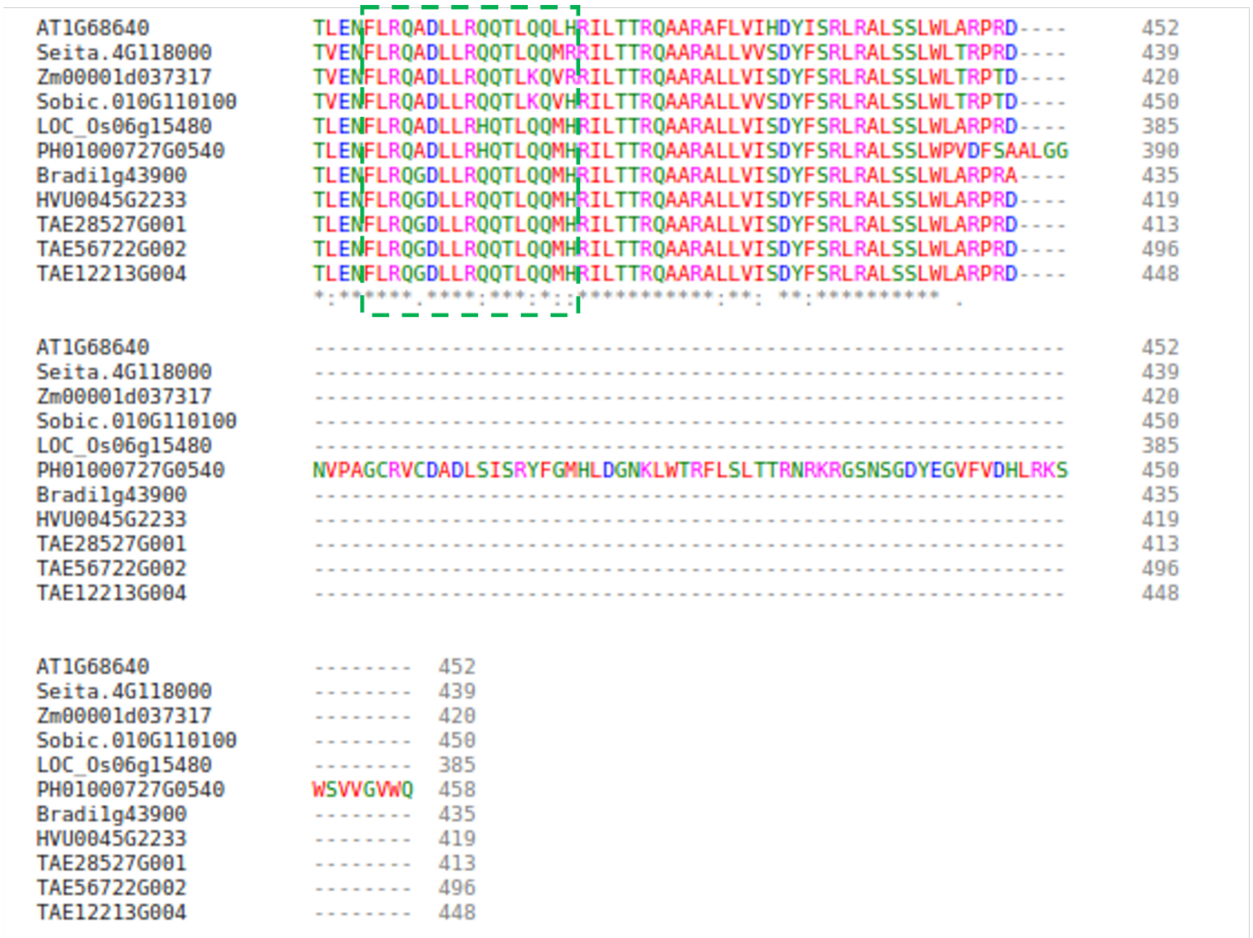


**Supplemental Figure S8.** Multiple Sequence Alignment of *OsbZIP47* orthologues. The sequence analysis of OsbZIP47 and its orthologs from millet, maize, sorghum, bamboo, brachypodium, barley and wheat was performed by Multiple Sequence Alignment. The basic leucine zipper DNA binding domain is marked in black box from 93AA-115AA, leucine zipper domain is highlighted in blue box from 116 AA-186 AA and the two transactivation domains are highlighted in gray and green box from 262 AA to 293 AA and 335 AA to 349 AA, respectively. Conserved and non-conserved cysteine (Cys) amino acids are indicated with red arrowhead and yellow shading. The amino acid number used to refer to protein domains and to refer to Cysteine residues are as per the position in the rice protein OsbZIP47.

**Supplemental Figure S9**

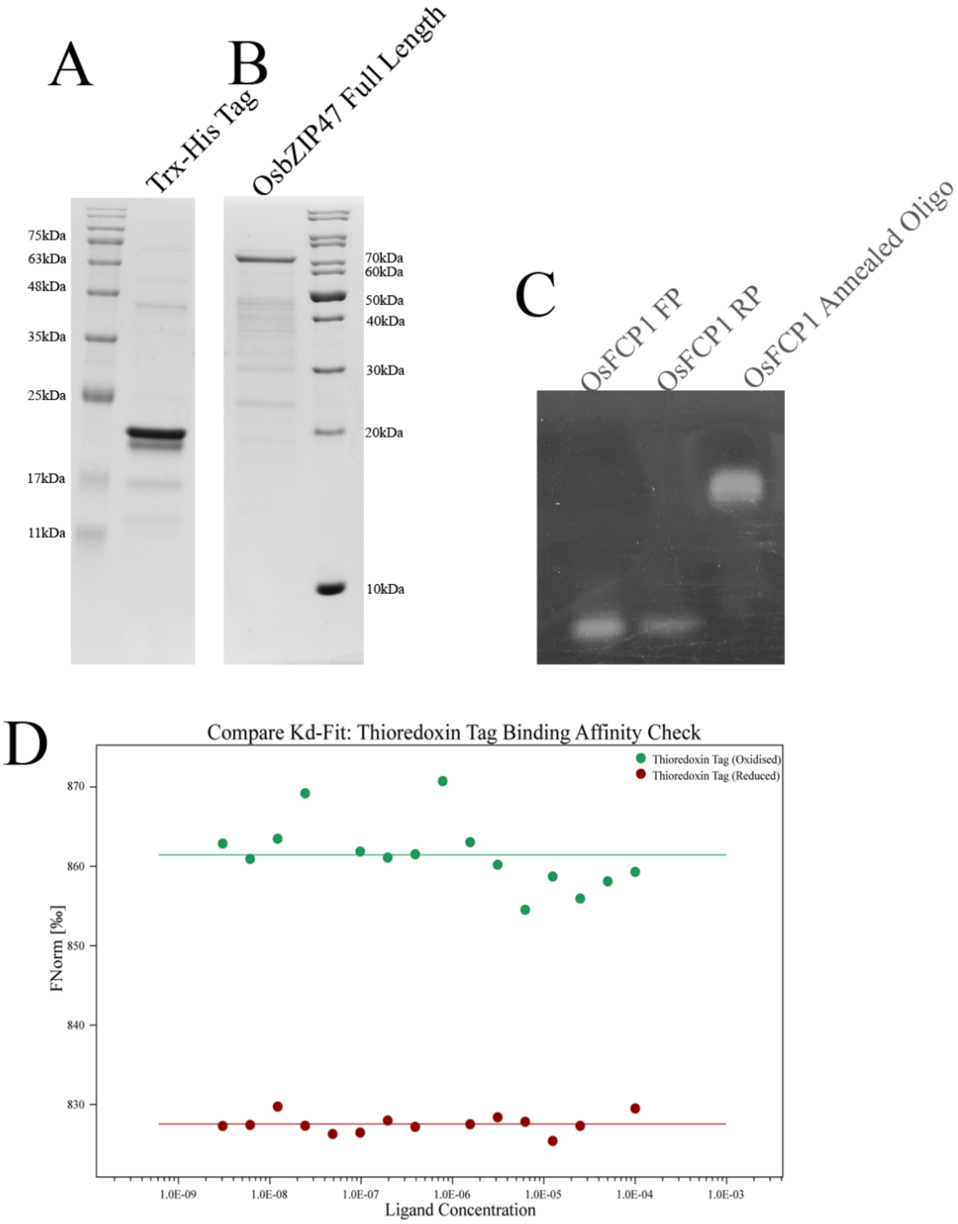


**Supplemental figure S9:** OsbZIP47FL purified protein and the control Trx-His tag protein alone used for DNA protein interaction analysis, and MST assay with the annealed double-stranded OsFCP1 oligonucleotide. **(A)** and **(B)** The 14% SDS PAGE gel images show the purified Trx-His tag alone protein and the purified OsbZIP47FL protein. The DNA protein interaction analysis carried out for tag alone is shown here as control. **(C)** Single-stranded forward and reverse DNA oligonucleotides from OsFCP1 locus (TGACGT cis motif located at -371bp of OsFCP1) run on

8% native PAGE gel along with annealed double-stranded OsFCP1 oligonucleotide. **(D)** Microscale thermophoresis experiment with the Trx-His tag alone done as control. A flatline with comparable fluorescence intensity at all concentrations of OsFCP1 oligo indicates no binding by the Trx-His protein tag alone.

**Supplemental Table S1.** Phenotypic quantification of *OsbZIP47KD*#14 of T3 generation plants

| Trait | **TP309WT/ Mean ± SEM** | **dsRNAibZIP47/ Mean ± SEM #14** | **P-value** |
|---|---|---|---|
| Seedling height (8 DAG, cm) | 5.90 ± 0.17 (N=38) | 4.816 ± 0.1 (N=31) | **** (< 0.0001) |
| Days to floral transition (days) | 53.57 ± 1.5 (N=14) | 75.83 ± 1.3 (N=12) | **** (< 0.0001) |
| Plant height at booting (cm) | 22.30 cm ± 0.6 (N=25) | 17.39 ± 0.5 (N=10) | **** (< 0.0001) |
| Lamina angle ($^0$) | 12.62$^0$ ± 1.3 (N=10) | 45.84 ± 5.3 (N=10) | **** (< 0.0001) |
| Panicle length (cm) | 9.71cm ± 0.3 (N=13) | 8.189 ± 0.3 (N=9) | ** (0.0056) |
| No. of primary branches | 3.1 ± 0.2 (N=9) | 2.0 ± 0.2 (N=12) | ** (0.0015) |
| Number of spikelets | 22.1 ± 1.3 (N=13) | 16.6 ± 1.4 (N=9) | * (0.0133) |
| Seed size (Length/Width ratio mm) | 1.85 mm ± 0.03 (N=43) | 1.643 ± 0.0 (N=41 | **** (< 0.0001) |
| SAM area (μm$^2$) | 996.5 ± 54.44 (N=12) | 1262 ± 52.25 (N=15) | ** (0.0018) |
| SAM width (μm) | 47.87 ± 1.68 (N= 10) | 52.99 ± 1.33 (N=12) | * (<0.05) |
| SAM height (μm) | 34.84 ± 0.93 (N=10) | 36.32 ± 0.95 (N = 12) | ns (>0.05) |
| Number of cells in L1 layer | 18 ± 0.58 (N=5) | 14 ± 0.49 (N=5) | *** (<0.0008) |

| Supplementary Table S2 : List of oligos used in this study | |
|---|---|
| **Name** | **Sequence 5'→3'** |
| **Primer for qRT-PCR** | |
| OsbZIP47 FP RT | GAG GGA CTA GAC TGA CTA AAG AG |
| OsbZIP47 3'UTR RP | CTC CTT TTG ACA TTG TAC GCG G |
| OsFON2 RT FP2 | ATG GGC CGG CTC TTC TTG TG |
| OsFON2 RT RP2 | CCT GCG CTC CAA TCA AAC GAC |
| OsFCP1 RT FP2 | ATC GTC GTC ACA ACC AGC GG |
| OsFCP1 RT RP2 | TCG TCG TCG TCG TCG TGG T |
| OsCYP734A2 RT FP | ATGTGCGTAGGCCAGAACCT |
| OsCYP734A2 RT RP | GGTATAGGAGCATCAGCACTGTC |
| OsCYP734A4 RT FP | GACAGCGGCTTCAACGACTT |
| OsCYP734A4 RT RP | AGGTTGGTGGTCGTCTGCTT |
| OsCYP734A6 RT FP | GTTCATACCGTTCGGCCTTG |
| OsCYP734A6 RT FP | GACGTATCTGGCCGACAACC |
| Os01g56330 FLR2 RT FP | 5AAC AGT CTT TGC TTG TCA CCT GC |
| Os01g56330 FLR2 RT RP | AAT GAA GAT GGT CTT GCG CAT AGC |
| Os01g0356951 lncRNA RT FP | GAC CGC GAC ATG CAT CAC CTC |
| Os01g0356951 lncRNA RT RP | GCA ACA CTC GCT CTA CTC GGC |
| OsCCT33 FP | TAG GGC TGT CCA AAC TTC AGG AGA |
| OsCCT33 RP | GAC AAA CTC TAC AAT GTA CGA GGT CC |
| OsFKF1 FP | GAA GCC GAC ATG GAG GAT TTT GAA CG |
| OsFKF1 RP | TTG GGA ATC ACT CAT CTT CGT CGG G |
| OsSPL18 FP | GCA CCA CCA CCA CCA CCA AAT CC |
| OsSPL18 RP | TGC TGC TGC TGC TGC TCG CTG T |
| OsCUC1 FP | GACTTCTGGAACTAATCC |
| OsCUC1RP | AAGGAGGAAGAGAAGGAT |
| OsAPO1 FP | TAA ACGGATGAACCGAGGAT |
| OSAPO1 RP | AAAGGGGAGGCCCATCTGAT |
| OsMADS16 FP | 5'-CCAAACCACTTGATGTGTGC-3' |
| OsMADS16 RP | CCAAGCACAGTTGCACAC |
| OsYUCCA6 FP | CCA TTC CCA GAT GGT TGG AAG G |
| OsYUCCA6 RP | CAT GTT GCG CCT CAAGATATTTG |
| OsYUCCA7 FP | GGA GGT GCA TCT CCG TCA TCT TC |
| OsYUCCA7 RP | CAC TGC TGT GTC CTA CAA TAT CAC |
| UBQ5 FP | ACCACTTCGACCGCCACTACT |
| UBQ5 RP | ACGCCTAAGCCTGCTGGTT |
| | |
| **Oligos for OsbZIP47 EMSA** | |
| OsFCP1 locus EMSA-371_FP | 5'-CAG CTG GCC CTG ACG TCA GCA ACC GG-3' |
| OsFCP1 locus EMSA-371_RP | 5'-CCG GTT GCT GAC GTC AGG GCC AGC TG-3' |
| | |

| Primers for cloning | |
|---|---|
| OsbZIP47 3'UTR FP | GGA TCA ATA TCA TTT GCC TGA GTT CT |
| OsbZIP47 3'UTR RP | CTC CTT TTG ACA TTG TAC GCG G |
| OsbZIP47 FL 5' FP (BamHI) | GGATCCATG GCG GCG CAC CAG GGG ATG |
| OsbZIP47 FL 3' RP | TAG TCC CTC GGC CGC GCC AG |
| OsbZIP47Δ582-1.14 CDS RP | GAC GAG CTC GCA CAG CTC GTC GT |
| OSH1 CDS FP | CTCATGGAGGAGATCTCCCACCA |
| OSH1 CDS RP | AGGCCGCCGTCGTTGATGAACT |
| RFL CDS FP | AGATCTCCCGGGATGGACTACAAAGACATGACGGT |
| RFL CDS RP | AGGCATCGTTGGGATCCTTGTCATCGTCATCCTT |
| OsMADS1 M FP (BamHI) | GGATCC ATG GGG AGG GGG |
| OsMADS1 KC14 RP | TAG TAA TTC CTG TAA CTT TTT CCT CAA GTC |
| OsMADS15 K FP (BamHI) | GGA TCC GAG ATA ACT CTT CCC CAG TTG ACA |
| OsMADS15 C RP | TTA AGC ATT GAG GTG GCT CAG CAT |
| OsETTIN1 FP | CCGGAATTCGGGATGACCGGGATCGACC |
| OsETTIN1 RP | TGAAATATCAATCACCCTGCCAACAACAGGC |
| OsETTIN2 FP | CCGGAATTCCGGATGCGGGAGGGAGAGGAC |
| OsETTIN2 RP | CGGGATCCCGTCAGATCATCGTATTCACTGCTG |

**Supplemental Materials and Methods:**

**RNA *in situ* hybridization**

To generate H4, Histone riboprobe, a PCR amplified gene-specific 229bp DNA fragment joining the 3'UTR with the end of the coding sequence of LOC_Os10g39410 was cloned in pBluescript KS+ vector. The resulting plasmid pBSKS H4 ISH#3 was linearized with HindIII and transcribed with T7 RNA polymerase to make sense RNA probe. For anti-sense riboprobe, the pBSKS H4 ISH#7 plasmid was linearised with HindIII and transcribed with T7 RNA polymerase. Tissue processing and probe hybridizations was done as described in Prasad et al. (2005). Signal was developed using anti-digoxygenin-alkaline phosphatase (AP) conjugated antibodies (Roche) and BCIP (5-Bromo-4-chloro-3-indolyl phosphate), NBT (nitro blue tetrazolium) chromogenic substrates (Roche).

**ONPG assay**

To examine and compare the strength of protein interactions in the yeast two hybrid assay, quantitative ß-galactosidase activity was determined with substrate 2-nitrophenyl ß-D-galactopyranoside (ONPG, Sigma). Single yeast colony was inoculated in YPD media and grown at 30 °C, overnight. Cells with $OD_{600}$ 0.5–0.7 were pelleted and resuspended in Z-buffer (60 mM $Na_2HPO_4$, 40 mM $NaH_2PO_4$, 10 mM KCl, 1 mM $MgSO_4$, pH 7.0) containing ß-mercaptoethanol as well as 4 mg/mL ONPG solution. The reactions were incubated at 30 °C and stopped after 30 min for the development of yellow colour by adding 1 M $Na_2CO_3$. Absorbance was measured at 420 nm, and ß-galactosidase activity was calculated according to the following formula: ß-galactosidase activity = $1000 \times A_{420}/(T \times V \times A_{600})$, where T = time of reaction (min), and V = volume of culture used in the assay (mL).

**Multiple Sequence Alignment**

Proteins homologous to OsbZIP47, from the indicated species, were downloaded from Plaza (Dicot 3.0 and Monocot 3.0) database. Multiple Sequence Alignment (MSA) was performed for proteins from Arabidopsis, Millet, Maize, Sorghum, Rice, Bamboo, Brachypodium, Barley, and Wheat using MUSCLE algorithm on the EMBL-EBI search and sequence analysis tools APIs in 2019 platform with default parameters.

**Non-reducing SDS-PAGE analysis**

Redox dependent OsbZIP47 oligomerisation was analysed as described in Gutsche and Zachgo (2016). 10µg OsbZIP47-Trx tag bacterial protein was treated with either 20 mM DTT (reducing agent) or 2 mM diamide (oxidative agent, S-glutathionylation), or first 2mM diamide treatment for 30minute followed by 20mM DTT treatment for 30minute at RT. Free cysteines were blocked by 10mM Iodoacetamide (IAA) and 15min incubation in dark at RT. Protein pellet of each condition was resuspended in 1x Laemmli-buffer (without any reducing agent) and boiled for 5 min at 95°C. Protein samples were loaded on 12% SDS-PAGE gel and bands were visualised using Coomassie Brilliant Blue staining. EMSA and MST experiments were performed as detailed in materials and methods section of the main text.

**Gene Ontology (GO) enrichment and meta-analysis**

To identify functional categories of differentially expressed gene sets (positively and negatively regulated by OsbZIP47), GO enrichment analysis was done using GOMAPMAN (http://www.gomapman.org/ontology) database as reference. The results are represented as positive and negative networks. The differential enrichment of different members of transcription factor classes and of various hormone metabolism and signaling genes was also determined and represented as heatmap. The significance of enrichment or depletion was calculated with 'Phyper' function in R package. The resultant p-values were used to construct the heatmap and enrichment networks. The genes deregulated by RFL, OsMADS1 and OsbZIP47 were compared and are provided in Supplementary dataset 2. The genes targeted by OSH1, bound in ChIPseq dataset from (Tsuda et al., 2014) were also identified. The quantitative representation of distinct and common list of genes between the three datasets is shown as Venn diagram.
